# Systematical Detection of Significant Genes in Microarray Data by Incorporating Gene Interaction Relationship in Biological Systems

**DOI:** 10.1371/journal.pone.0013721

**Published:** 2010-10-29

**Authors:** Junwei Wang, Meiwen Jia, Liping Zhu, Zengjin Yuan, Peng Li, Chang Chang, Jian Luo, Mingyao Liu, Tieliu Shi

**Affiliations:** 1 The Center for Bioinformatics and Computational Biology, The Institute of Biomedical Sciences, The School of Life Sciences, East China Normal University, Shanghai, China; 2 The College of Financial and Statistics, East China Normal University, Shanghai, China; Texas A&M University, United States of America

## Abstract

Many methods, including parametric, nonparametric, and Bayesian methods, have been used for detecting differentially expressed genes based on the assumption that biological systems are linear, which ignores the nonlinear characteristics of most biological systems. More importantly, those methods do not simultaneously consider means, variances, and high moments, resulting in relatively high false positive rate. To overcome the limitations, the *SWang* test is proposed to determine differentially expressed genes according to the equality of distributions between case and control. Our method not only latently incorporates functional relationships among genes to consider nonlinear biological system but also considers the mean, variance, skewness, and kurtosis of expression profiles simultaneously. To illustrate biological significance of high moments, we construct a nonlinear gene interaction model, demonstrating that skewness and kurtosis could contain useful information of function association among genes in microarrays. Simulations and real microarray results show that false positive rate of *SWang* is lower than currently popular methods (T-test, F-test, SAM, and Fold-change) with much higher statistical power. Additionally, *SWang* can uniquely detect significant genes in real microarray data with imperceptible differential expression but higher variety in kurtosis and skewness. Those identified genes were confirmed with previous published literature or RT-PCR experiments performed in our lab.

## Introduction

DNA microarray technologies have been widely used in biological studies, and simultaneously measure expression levels of thousands of genes across cells or tissues under different conditions [1]. In the microarray data analysis process, one of the most important steps is to determine whether a gene is differentially expressed under particular conditions since follow-up analysis depends on the selected differentially expressed genes (DEGs). Nevertheless, the selection of the DEGs is associated with both statistical and biological problems [2]. The biological problem is whether the identification of DEGs should consider nonlinear biological system. Practically, gene interactions are nonlinear [3]–[5]. In nonlinear systems such parameters (mean, variance, skewness, kurtosis) can be interdependence [6], where skewness and kurtosis are defined as nonlinear index [7] and can be preserved even in a weakly nonlinear network or system [8], [9]. When an input signal follows normal distribution, nonlinear system (e.g., quadratic) can produce an output signal with non-Gaussian distribution [7], [9]. Hence, skewness and kurtosis should be used in evaluating nonlinear systems. Statistically, some current existing tests for DEGs detection assume linear relationships, Normal distribution, and large sample sizes according to the classical statistics. In fact, the limitation of resources and high cost of the microarray experiments make the sample sizes usually much smaller relative to the number of considered genes, which results in the decrease of the statistical power (SP), high false positive rate (FPR), and the enlargement of sample's error [10].

Many methods, such as T-test, SAM [11], two-sample Bayesian T-test [2], and Fold-change, have been proposed to detect DEGs according to the location (mean) difference of case-control. T-test is a classical and useful statistical method but it can only detect the different means of gene expression profiles. SAM, a derivation of T-test, uses the same principle as T-test to detect DEGs and its uncertainty s_0_ has significant effects on the mean difference detection of gene expression [1]. Similarly, the principle of Golub's discrimination score [12], Welch t-statistic [13], t-type score [1], probe level locally moderated weighted median-t (PLW) [14], and locally moderated weighted-t (LMW) [14] focus on the difference of locations. Two-sample Bayesian T-test [2], which can be used for the small sample size via incorporating prior information, still detects DEGs based on the mean difference. Finally, Fold-change [15] is a simple method to detect the mean difference of gene expression. However, all of those methods are unable to use the information of variance, kurtosis and skewness of gene expression simultaneously.

In contrast, other methods, such as Hartley, Cochran, and Bartlett test, could utilize sample variance difference to detect DEGs. These methods identify DEGs without considering the difference between means based on an assumption that the logarithm of expression-level measurement of a gene under a given condition has a rough Gaussian distribution. Meanwhile, nonparametric methods without any distribution hypothesis have also been used to select differentially expressed genes, but much information is ignored because those methods only considerate the rank of samples.

Alternatively, other methods have been developed to detect DEGs on the basis of large-scale data, or statistical models. The false discovery rate (FDR) [16], ranking analysis of microarray data (RAM) [17], FDR-base methods [17], and optional discovery procedure (ODP) [18] identify DEGs through ranking the statistics of any statistical method based on large-scale data. FDR, an expected proportion of the false positive among all the positives detected, is to control the erroneous rejection of a number of true null hypotheses, while RAM is a large-scale two-sample t-test method and is based on the comparisons among a set of ranked T statistics. Hence, the first step of FDR and RAM is to calculate statistics of each gene in a microarray. In the application of ODP, the assumption of the null distribution and alternative distribution is the prerequisite. Hotelling' T^2^ test is to test the different mean vectors of entirety genes of case-control, yet it is still limited by the smaller sample sizes relative to the number of considered genes. The MFS-Hotelling' T^2^ [19] is not affected by sample sizes but is still based on means and covariance. Those methods are designed for large-scale data, while other methods based on statistical models have been proposed, like Bayesian method included probe-level measurement error (BPLME) [14], and F_S_ test [20]. BPLME employs Bayesian hierarchical models to estimate probe-level measurement error which is utilized to adjust the variance for selecting DEGs. Similar to ANOVA and F-test, F_S_ test is based on generalized linear model to estimate shrinking variance to determine DEGs. Although these methods may be robust in finding DEGs to a certain extent, they all ignore the information of the high moments.

Unlike other methods, ANOVA, a generalization of the *t*-test, allows for the comparison for more than two conditions' samples. Similarly, F-test, fixed-ANOVA, and mixed-ANOVA are designed to detect DEGs under several conditions [21]. However, they only consider the information of the locations.

In all, current published methods are adjusted basic statistics methods and try to decrease the FPR in microarray analysis according to aforementioned approaches. Those methods only focus on difference of location or variance and ignore the difference of high moments, which could possibly lead to error in certain parts of randomization theory [22]. Moreover, they also ignore the functional association from those functionally related genes in microarray experiments because they assume that biological systems are linear and their approaches follow Normal distribution, respectively. During the process of DEGs selection, those statistical methods simply discard the genes which may actually be quite important because they display insignificance in different means or variances between case and control. Therefore, those methods normally have comparatively low statistics power with high FPR [10]. It is still a challenge that how to improve SP and maximally extract the useful information from the microarray data by incorporating the information about the functional relationship between genes from the microarray data with relatively small sample size [10].

To decrease FPR and improve SP, we present the *SWang* test to detect the DEGs not only by utilizing means, variances, skewness, and kurtosis simultaneously, but also by recognizing and latently incorporating the functional relationship of genes in biological systems. In the study, we conduct comparative evaluation of the performance between *SWang* and other tests, like T-test, F-test, SAM, and Fold-change, based on simulated and real microarray data. Two real microarray datasets of breast cancer are employed to test *SWang* method and other four tests. Moreover, we carry out experiments at the bench to confirm those genes uniquely identified as being differentially expressed by *SWang*(1,4). All the results demonstrate that our method is superior to the other four statistics methods for the DEGs detection.

*SWang* test has several unique characteristics compared to the current popular methods.

First, *SWang* utilizes the information of multiple and high moments which have been used to summarize the shape of a probability distribution in probability theory (**File S1 text**). The high moments represent certain information of distributions, e.g., the skewness indicating symmetric distribution. The positive skewness means the asymmetric distribution with longer right tail while negative skewness indicates the asymmetric distribution with longer left tail [23]. Therefore, the first moment (also known as mean) could not be enough to represent all the location information in asymmetric distribution. The positive kurtosis means that most of the variance is the result of infrequent extreme deviations, as opposed to frequent modestly sized deviations [23]. To illustrate the biological significance of high moments, we construct a nonlinear gene interaction model to demonstrate that high moments contain the information of association among genes. Although the estimated high moments may be biased, the estimation of kurtosis could be reliable in Pearson' distribution family with relatively small sample size [23] and they are necessary to be considered in detecting DEGs. Because the sample size is much smaller than the size of genes under most circumstances in the microarray application, and the small sample size makes the law of large number invalid [24], [25], it indicates that the mean and variance contain insufficient information of the data when the sample size is small.

Second, we assume that the distribution of gene expression profile belongs to Pearson distribution family that includes normal distribution, exponential distribution, Gammas distribution, or mixture of Gaussian/Gammas distribution, according to previous studies [25]–[27].

Third, from the statistical view, the highest moment for the samples should be four and the fourth moment corresponds to kurtosis [22]. Our method realizes and utilizes multiple moments simultaneously, since it can also be statistically proven that the high moment is necessary and essential for the gene differential expression detection under the small sample size.

Fourth, *SWang* latently incorporates the biological facts that functionally related genes have effects on the expression levels of one another. Although the associations among genes are not easy to be estimated, they could be recognized via considering all moments according to nonlinear gene interaction model and nonlinear biological system.

Finally, *SWang* can be used to detect DEGs with different combinations of different moments which depend on the sample size and the assumption of distribution. *SWang* is based on a null hypothesis that the mean, variance, skewness, and kurtosis between case and control should be equal. Although there are total of 15 combinations, we suggest that it is better to consider the four different moments simultaneously during the application.

## Results

To evaluate the performance of *SWang*, we carried out two statistical simulations to measure and compare the FPR and SP under Pearson distribution family between *SWang* and four other methods, including T-test, F-test, SAM(s0 = 0.3), Fold-change. The first simulation was to calculate FPR without considering gene associations under various distributions and the second was to measure SP with the consideration of nonlinear biological system. Next, DEGs were selected in real microarray data with those methods. During the processes, the criteria for p-value and q-value are 0.05 (or 2-fold change), and these rigorous criteria are to minimize the false positive results [28]. Subsequently, the results generated with those five different methods are compared with each other. Additionally, we also use ‘spike-in’ data to evaluate the *SWang*.

Firstly, we randomly drew samples from both case and control groups. The distribution of case group was considered as exponential, normal, uniform, cauchy distribution, complex of triangular, normal, and exponential distribution, or mixture of normal distribution, respectively, when the distribution of control group was regarded as normal distribution with mean as 1 and standard deviation as 1.5. Sample size was the combination of the sample size of both case and control from 3 to 53 (Details in **File S4**). This step is to generate a pair of case and control groups for a gene of interest to test whether the gene is differentially expressed. During simulation, we randomly assigned 20% genes as DEGs to calculate false positive rate when the distributions of case and control are the same and explore the differences of skewness and kurtosis between case and control from either small samples or real gene-expression variation. Then we calculated the p-value of *SWang*, T-test, SAM, and F-test or fold-change ratio. Subsequently, we counted the number of those genes with *p*-value less than 0.05 or fold change greater than 2. Finally, we calculated the FPR and SP of each method [29].

Next, we drew the figures with false positive rate as vertical coordinate and cut-off p-value as horizontal coordinate according to the simulation results. The cut-off p-value is the theoretical false positive rate which is the ratio of undifferentially expressed genes selected as DEGs to the total number of DEGs in theory. The false positive rate is the real p-value generated from the simulation results. Practically, if the curve in generated figures is above diagonal line, it indicates that the real false finding ratio is higher than estimated false finding ratio, and the method is unconvincing, so that the result obtained by this method is undesirable with low confidence. In contrast, if the curve is on or below the diagonal line, the real false finding ratio is equal to or less than the estimated false finding ratio, resulting in the satisfied findings with high confidence.

**Figure 1:**
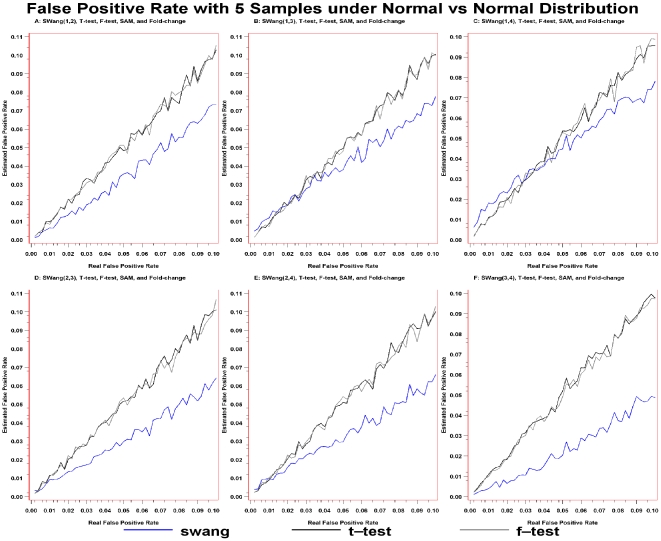
False positive rate of T-test, F-test, Fold-change, SAM(0.3), and *SWang* with 5 samples for both case and control from Normal distribution. A: False positive rate of *SWang(1,2)* and other methods. B: False positive rate of *SWang(1,3)* and other methods. C: False positive rate of *SWang(1,4)* and other methods. D: False positive rate of *SWang(2,3)* and other methods. E: False positive rate of *SWang(2,4)* and other methods. F: False positive rate of *SWang(1,3)* and other methods. The false positive rate of T-test(black spotline), F-test(gray spotline), Fold change(not shown), *SWang*(blue spotline), and SAM(0.3)(not shown) with cutoff of p-value. Note that the curves of SAM and Fold-change cannot be drawn due to false positive rate of SAM and Fold-change that can not calculated, as all real DEGs are considered as non-DEGs under both methods.

The results showed that the curves of F-test, T-test, and *SWang* displayed lower false positive rate with their curves on or below the diagonal line, with the curve of *SWang* located at lowest level (**Figure 1**).

Traditionally, the microarray data are regarded to follow normal distribution. We tested the performance of *SWang* and others under this assumption. Comparing the curves generated from other methods, the slope value of the curve for *SWang* with different combinations of moments and different sample sizes are the smallest. It can be seen that the separation ability for the curve between *SWang* and others is largest when fourth moment (kurtosis) is applied in *SWang* to detect DEGs. Simulation results also show that the FPRs of *SWang* with high moments are the lowest among all the tested methods, regardless of the sample size (**Figure S4**).

In fact, the real microarray data distribution does not always fit normal distribution. Therefore, we considered the situation that the distribution of microarray data belongs to Pearson distribution family, and also tested the performance of *SWang* and other methods under various Pearson distributions. Exponential distribution is a special gamma distribution which is a subset of Pearson distribution family. The curves generated from those methods indicate that the slope of the curve for *SWang* is the most gradual. The FPR of *SWang* with high moments are the lowest with sample size greater than 5 (**Figure S6**). We obtained the similar results on other Pearson distribution including Uniform distribution, and Cauchy distribution.

The results from **Figure 1**, and **Figure S1**, **S2**, **S3**, **S4**, **S5**, **S6**, **S7** demonstrated that our methods have good performance with higher confidence in DEGs detection, and that the differences of skewness and kurtosis between case and control are not due to the sample size but to real gene-expression variation. When high moments are utilized, FPRs of *SWang* are less than those of other methods. As the sample size increases, the significance of skewness and kurtosis correspondingly decreases.

We also verified the effectiveness of *SWang* with the ‘spike-in’ data [30] that contain a limited number of spiked-in cRNAs. The ‘spike-in’ data is a control dataset which has been used for evaluating the effectiveness of analysis methods for microarrays. This dataset has several features to facilitate the relative assessment of different analysis options [30]. Our analysis demonstrated that the *SWang*(1,4) option provided the lowest false positive rate (**Figure S8**). The other options of *SWang* proved less effective compared to the SAM and Fold-change methods, this could be due to the criteria in ‘spike-in’ experimental design for selecting DEGs solely based on Fold-change. In addition, the experimental design for the ‘spike-in’ data may not even have considered gene function associations.

**Figure 2:**
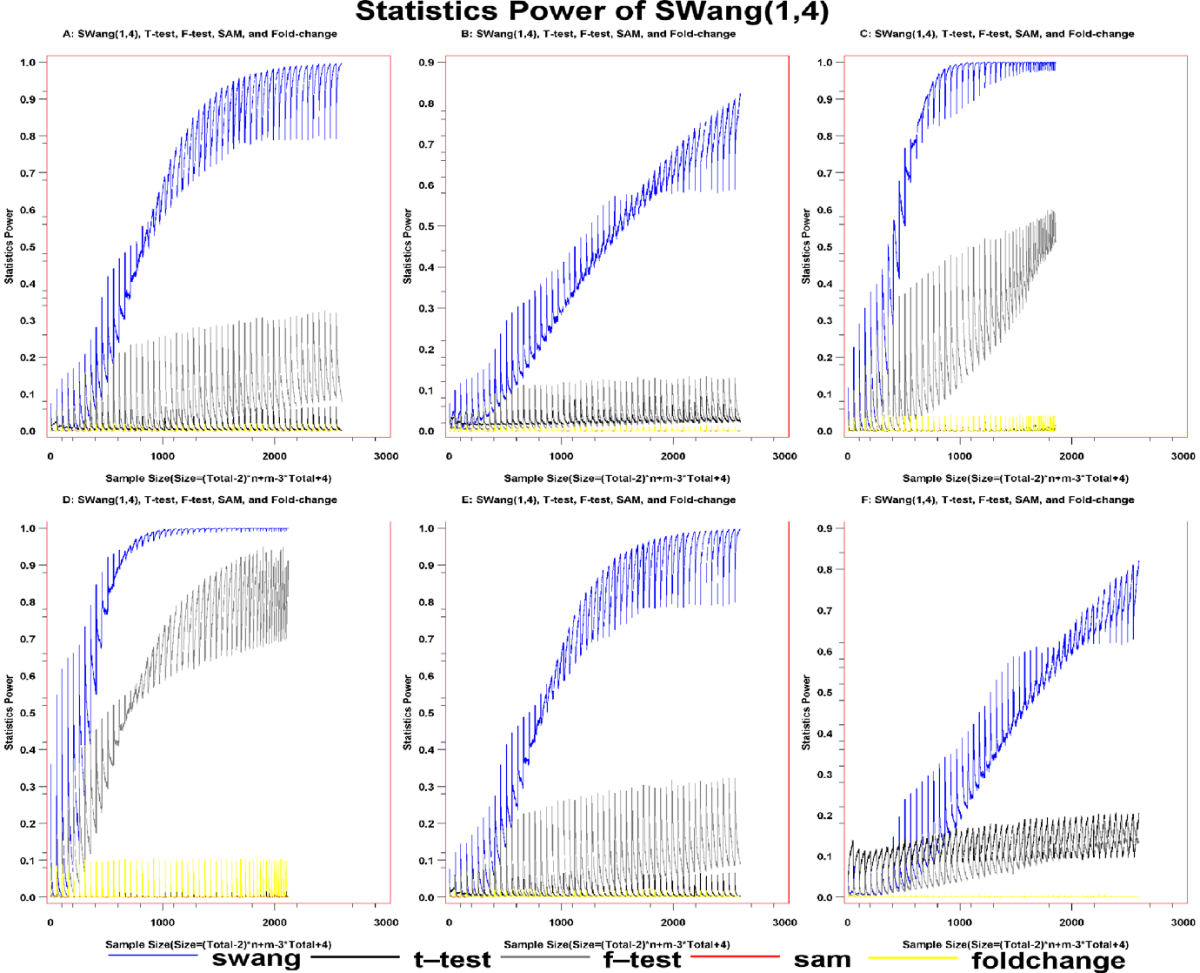
Statistical power of T-test, F-test, Fold-change, SAM(0.3), and *SWang*(1,4). The distributions of control are normal, Statistics power of those methods are A: under Normal distribution for case. B: under Exponetial distribution for case. C: under Uniform distribution for case. D: under Gamma distribution for case. E: under mixture of gamma and normal distribution of case group. F: under complex distribution which is a combination of various distribution for case. The statistics powers of T-test(black spotline), F-test(gray spotline), Fold-change(green spotline), *SWang*(blue spotline), and SAM(0.3)(red spotline), while the size in the coordinate is equal to the sample size of control group times that of case group with power as 0.2.

We then evaluated the robustness of *SWang* with simulation when considering nonlinear biology system (**Figure 2**, and **Figure S8**, **S9**, **S10**, **S11**, **S12**, **S13**, **S14**, **S15**). The sample size is the combination of the sample sizes from both case and control groups and the SP is the ratio of the number of DEGs to the total genes generated from the simulation results. Visually, the far the curve in the figures from the horizontal coordinate, the better the method.

Under normal distribution, the curve of the *SWang*(1,4) option is on the top, the curve of F-test is the second, below that of *SWang*(1,4), and the curves of the other three methods separate from that of *SWang* and F-test significantly, being close to baseline (**Figure 2A**). More interestingly, under Exponential distribution, the curve of *SWang*(1,4) is on the top and far apart from the curves of other four methods (**Figure 2B**). Similar results can be observed under Uniform, Gamma, mixture of Gamma and Normal, and complex distribution (**Figure 2C–2F**). When the sample size is very small, it seems that the curves of *SWang* and the other four methods cannot be distinguished. To further compare SP between *SWang* and the other four methods under small sample size, we drew **Figure S9** with small sample size and the result illustrates that the curves using the *SWang*(1,4) option are still on the top. Therefore, it is shown that SP of *SWang* is always larger than other methods under various tested distributions, indicating that *SWang* has the best performance among those five methods.

To compare SPs under different moment combinations of *SWang* with the other four methods, we also measured SPs with Pearson distribution family. Under normal distribution, the curves of *SWang*(1,3) and *SWang*(1,4) are farther from the horizontal coordinate (**Figure S10**), demonstrating that the SPs of *SWang*(1,3) and *SWang*(1,4) are larger than those of the other four tested methods. F-test has the second largest SP under the test situation. Although there are some overlap between curve of *SWang* and the others methods, the curves of *SWang* are higher that those of the others when considering small sample size (**Figure S11**). Simulation results under other Pearson distribution family also showed that the curves of *SWang*(1,3) and *SWang*(1,4) to be far above the curve of other methods (**Figure S12**, **S13**, **S14**, **S15**, **S16**). These analyses demonstrate that SPs of *SWang*(1,3) and *SWang*(1,4) are larger than those of the other methods, and that it is necessary to utilize high moments to detect DEGs.

To evaluate the performance of our method on real data, we used the *SWang* (1,4) option to detect the DEGs in both dataset1 and dataset2 related to breast cancer (See Material & Method), and then mapped those selected genes to human biological pathways based on the KEGG system. Here, we only focus on those genes that are mapped to the cancer related pathways from KEGG.

In Dataset1, the previous study has identified 160 significantly differentially expressed genes at threshold of *q* values≤0.05 [28]. In the same dataset, our method detected 157 genes with differential expression in those 160 genes. The three genes that are not selected by our method all have higher *p*-value in our result, although the *q*-value for those genes is less than 0.05 based on previous method. The *p*-value for gene CKS2 (Mutation Id 359119) with our method is 0.05667 when its *q*-value is 0.04540; gene MYCLK1 (Mutation Id 417226) has a *q*-value of 0.04723, but its *p*-value based on our method is 0.05011; meanwhile, the Mutation Id (HV18H8) corresponding to an unknown gene is also detected as differentially expressed with a *q*-value of 0.04984 based on previous study, but in our result its *p*-value is 0.055762, larger than 0.05.

We also applied several other common different statistical methods to detect the DEGs in both dataset1 and dataset2, and the overlapping genes selected by all of the applied methods are shown as 5-venn diagram in **Figure S18** and **Figure S19**. A number of genes were not regarded as significantly differentially expressed by other applied methods since their *p*-values were greater than 0.05 based on T-test, SAM(0.3), F-Test, or their fold changes were less than 2. Among these genes, our method has additionally detected 42 genes (63 Mutation Id) in Dataset1, 312 genes (362 probes) in Dataset2 as differentially expressed. Although there are larger number of genes detected by F-test and T-test than that of genes selected by *SWang*, T-test and F-test have higher FPR with respect to non-Gaussian distribution of microarray [19]. Moreover, T-test and F-test ignore the nonlinear biology system. The inherent limitations of the two tests could bring about high positive result.

To further confirm the results of our analysis, we randomly selected 9 genes from those genes detected by T-test, F-test, SAM(0.3), or Fold-change but not detected by our method in Dataset2, and carried out the same RT-PCR experiment. Because some of the gene names do not exist in NCBI anymore and the RT-PCR for some other genes was unsuccessful, eventually, we got RT-PCR result for 3 out of 9 genes. The result shows that those three genes are not differentially expressed between the breast cancer lines and control (**Figure S20**).

In dataset1, we focused on those uniquely selected genes (**Table S1 in File S5**) that are involved in the related cancer pathways. The relative statistics for those genes are listed in **Table 1**. TP53 (GeneID: 7157) was one of gene selected which encodes a tumor suppressor, that has been widely recognized as an important protein in various carcinogenesis. It is one of the components in MAPK signaling pathway [31]. Changes in the TP53 gene greatly increase the risk of developing breast cancer [32], [33]–[35]. TP53-mutated breast cancers have been shown increased sensitivity to high-dose chemotherapy or dose-dense epirubicin-cyclophosphamide.

**Table 1:** The value of genes of different statistics.

Mutation_id	gene	q-val	F_C	T_T	pt	sam	psam	F_T	Pf	*SWang*	P_sw
UG4B8	BCR	0.6568	−0.18	0.542	0.298	0.263	0.398	4.775	0.328	3.7843	0.0435
HV7G7	CASP3	0.6533	−0.02	0.081	0.468	0.030	0.488	1.951	0.007	3.8086	0.0428
LO1E11	CCND1	0.4079	−0.06	1.165	0.132	0.1560	0.438	0.13	1.365	5.1738	0.0179
LO5H3	EGFR	0.4683	0.004	0.016	0.494	−0.006	0.502	2.939	2E-04	5.9013	0.0118
HV4C6	IL1R1	0.4788	0.351	0.977	0.173	−0.471	0.677	7.003	0.912	3.7972	0.0431
HV25H4	MCM4	0.3918	−0.38	0.996	0.169	0.450	0.313	7.639	0.994	4.5766	0.0258
HV5D11	MYD88	0.5618	0.043	0.228	0.412	−0.083	0.532	1.531	0.058	3.7185	0.0456
HV16G1	PDGFB	0.1694	0.786	1.744	0.052	−0.917	0.812	12.58	2.92	4.4912	0.0272
HV31E10	RRAS2	0.1957	−0.18	1.552	0.072	0.416	0.342	0.807	2.428	3.7161	0.0456
UG4C10	TAGLN	0.5245	0.818	0.655	0.262	−0.455	0.672	76.68	0.438	3.7843	0.0435
LO2D5	TP53	0.2422	−0.52	1.444	0.086	0.721	0.242	2.261	0.157	5.249996	0.017

The genes and their related mutation Id in Dataset1, q_val is the q_value of gene expression, F_C is Fold-change of gene expression, T_T is T-test value, pt is the p-value of T-test. Sam is the value of SAM(0.3), F_T is the value of F_test, pf is the p-value of F-test for gene expression. *SWang* is the value of *SWang* for gene expression, and P_sw is the p-value of *SWang*.

In dataset2, mapping those uniquely selected genes by our method to cancer pathways left 12 genes, BID (GeneID: 637), CCNE2 (GeneID: 9134) [36], DVL3 (GeneID: 1857), FGF7 (GeneID: 2252), FGFR1 (GeneID: 2260), FGFR2 (GeneID: 2263), FZD4 (GeneID: 8322), MAP2K2 (GeneID: 5605), PDGFB (GeneID: 5155), PGF (GeneID: 5228) [37], PML (GeneID: 5371), and WNT1 (GeneID: 7471) (**Table S2 in File S5**). Interestingly, we found that those genes, WNT1, FZD4, and DVL3 are enriched in the Wnt signaling pathway (**Figure S17**) [38]–[41]. WNT1 detected as down-regulated in the dataset2 has been reported to be involved in human breast neoplasma.

Among those uniquely selected DEGs with our method in dataset 1 and dataset2, There are three common genes, PDGFB (GeneID: 5155), MCM4 (GeneID: 4173), and MYD88 (GeneID: 4615). The Fold-changes of PDGFB, MCM4, MYD88 in dataset 1 are 0.786, 0.043, −0.38, respectively while the Fold-changes of those genes in Dataset2 are 0.408, 0.427, −0.0046. Interestingly, the trends of overexpression or underexpression for those genes are consistent between those two datasets. The upregulated MCM4 gene in our result is one of the genes involved in DNA replication and cell cycle, it has been reported that mutation in MCM plays a role in cancer development in mice and may increase breast cancer risk in humans [41]. MYD88, an adaptor protein which is known to mediate the signaling of toll-like receptor (TLR), has been reported to mediate IFN-γ- induced MAP kinase activation and PD-L1 expression. Previous research has confirmed that TLR is expressed in breast cancer [42]. Meanwhile, it has been shown that chemopreventive agents potentiate IFN-γ-induced PD-L1 expression in human breast cancer cells [43].

**Figure 3:**
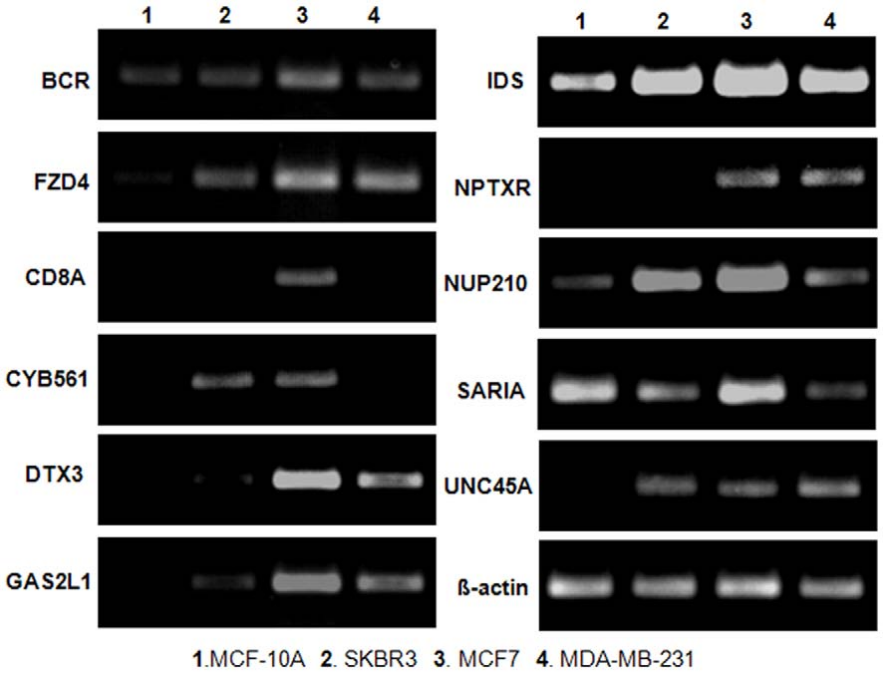
Semiquantative RT-PCR comparision. MCF-10A cells were cultured in DMEM/F12 with 10%FBS, 20ng/ml EGF, 0.5ug/ml Hydrocortisone, 0.01ug/ml Insulin and 0.1ug/ml Cholera toxin. MCF-7, SK-BR-3, MDA-MB-453 and MDA-MB-231 cell-lines were maintained in DMEM with 10%FBS. PCR products (MCF-10A, lane 1; SK-BR-3, lane 2; MCF-7, lane 3; MDA-MB-231,lane 4) were separated on 2% agarose gel and then stained with ethidium bromide. Stained bands were visualized under UV light and photographed. The beta-actin used as an internal control.

Finally, we utilized the same Semiquantative RT-PCR to verify those 12 genes uniquely detected with our method and not confirmed with experiments from published literature. The result clearly shows that those genes are differentially expressed between the different breast cancer lines comparing with the different metastasis abilities and the control (**Figure 3**).

## Discussion

A basic and crucial step in microarray data analysis is to detect DEGs from ten thousands of genes on microarray. Previously, several statistical methods [1], [2], [10]–[19] have been applied for the selection process, but the inherent biases of those methods limit their application and result in relatively high FPR [16]. Our proposed *SWang* test has the lowest false positive rate in simulations and the best performance using real microarray data to detect DEGs compared with those popular tested methods, because *SWang* latently considers the complicated gene interaction relationships acting on gene expression in biological systems and incorporates more concealed information of the microarray data, like kurtosis, skewness, and high moments which are ignored by other methods [20], [27]. Furthermore, results of SP in simulation indicate that *SWang* has comparatively significant performance whether or not the gene function association is considered.

In the microarray application, the nonlinear characteristics and small sample sizes always cause high FPR and low SP when detecting the DEGs with current popular methods. *SWang* incorporates skewness and kurtosis, and those moments can indicate nonlinear effects that should not be neglected when evaluating data with small sample sizes. Previous researches [7]–[9] and our analysis of nonlinear gene interaction model suggest that skewness and kurtosis can be used to measure nonlinear effects for nonlinear systems. Besides, according to the Law of Large Number, when the sample size is large enough, only considering the mean and variance could be enough to detect those genes with differential expression. However, under most of circumstances, the sample sizes in microarray experiments are too small compared with the number of genes found on the microarray, and the law of large number will become invalid. In such case, maximally using various information the data contains becomes more important to correctly select the DEGs and the curious property of moments of small sample size is that ignoring moments could possibly lead to error in certain parts of randomization theory [22]. *SWang* considers the high moments, and it yields both the lowest FPR and highest SP under the small sample size, compared with the other four methods.

For certain genes in a microarray experiment, even if the null hypotheses can be accepted when using T-test, F-test, or SAM, skewness and the kurtosis for those genes can be significantly different, indicating the distributions of both case and control are asymmetric and leptokurtic/platkurtic. Therefore, when the *SWang* statistical method is applied on the related data, the null hypotheses for those genes could get rejected. For instance, in dataset1, the statistics of T-test and F-test for gene BCR with fold-change −0.1775 are 0.5423 and 0.3282. However, the skewness and the kurtosis of the gene between case and control are larger than 1, with −1.6916, −0.6692 in case group, and −0.0750, 0.5691 in control group, respectively. In dataset2, the gene CD8A is regarded as not significantly differentially expressed based on the same popular statistics methods above, since its fold-change ratio is −0.002, with T-test value as −0.0047, and F-test value as 2E-05. However the skewness and the kurtosis of the gene are −0.4663, −0.7884 in the case group, and −0.2214, 0.6683 in control group, our method has recognized it as a gene with significantly differential expression, and the result is confirmed in the breast cancer cell lines with semiquantatitive-RT-PCR (**Figure 2**).

Although estimations of skewness and kurtosis of small sample size could be unstable, there is a stable way to extract information of skewness and kurtosis. The raw moments of any sampling distribution can be unbiasedly and separately estimated but they cannot take the expected values simultaneously [22]. However, for central-moments like mean, variance, skewness and kurtosis, and raw moments, the sample size should be greater than 4 when unbiased estimating the four central and raw moments, and the highest order of moments should not be greater than the sample sizes [22]. In addition, in the Pearson distribution family, reliable kurtosis can be estimated at relatively small sample sizes [23]. Furthermore, our simulation results for FPR also demonstrate that the sample size should be greater than 4 when estimating the first four moments of a distribution from Pearson distribution family. Finally, symmetric functions of raw moments are unbiased estimators of central moments [44]. As a result, to obtain stable skewness and kurtosis and avoid problem [44], we suggest to transform skewness with the third raw moments and kurtosis with the fourth raw moment.

The *SWang* test can have different combinations with different moments, and we can use *SWang* (*h,k*) to represent *SWang* test which includes information from *h*th moment to *k*th moment, where *h* is defined as greater than or equal to 1 and is the smallest moment, *k* is defined as greater than or equal to *h* and is the largest moment. Also, we can apply *SWang* test as the *SWang*((1,3,5)) format which means that *SWang* utilizes the information from the first, third, and fifth moments to test the difference between the case samples and control group. When *h* = 1 and *k* = 1, the *SWang*(1,1) is the square of classical T-test (**File S3**), as T-test has an assumption that the two-sample variances are equal. When k is greater than 2, the *SWang* test is not a general test. When all existing moments are employed in the *SWang* test, *SWang* will test whether the distributions of case and control are the same or not.

It should be noted that the *SWang* test is not an adjustment of T-test, because it can test the mean, variance, kurtosis, and skewness simultaneously. In contrast, T-test can only test the differences of mean without considering the differences of variance, kurtosis, and skewness. Since the function of Hotelling'T^2^ test (formula 10) can test multivariate simultaneously, our *SWang* method looks like the Hotelling'T^2^ test. However, our method can utilize the information of the moments that are from second moment to high moment when it is necessary according to the sample size and data.

*SWang* test can be used not only on datasets with large sample size but also on those with small sample size, the degrees of which depend on the sample size and the moment. The sample size determines how many moments need to be used, and conversely, the usage of the selected moment can also have an effect on the sample size. Practically, the highest moment should be four, because the underlying hypothesis distribution is normal distribution that belongs to the Pearson distribution family, which is supported by the characteristics of the gene expression. For the degrees of *SWang*(1,*k*), the sum of sample sizes from both case and control should be greater than *k*+1 and the minimum sample size of both case and control groups should be greater than or equal to 2. Otherwise, the *SWang* test will be invalid. Similarly, the total of samples from case and control should be at least six when using *SWang*(1,4) option. When the sample sizes of both case and control are equal to two, *SWang*(1,2) or *SWang*(1,1) should be adopted in the DEGs selection process. Strictly speaking, the sample sizes of case and control should be greater than 4 for *SWang(1,4)* for consideration of the reliability of skewness and kurtosis.

*SWang* can be used to detect DEGs based on the same distribution of null hypothesis which is transformed as 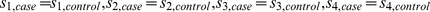 in Pearson family distribution. Realistically, the real distribution for microarray data is unknown and complex. Normally sample size is much smaller than the number of genes for microarray, thus the distribution for both case and control for all used datasets has been assumed to be exponential, log-normal, Gamma or their mixture distribution [26], [27]. However, those assumptions could be insufficient. Our results of Empirical distribution Test indicate that distributions of case and control for certain genes could be the same or different in the same dataset. For example, distributions between case and control for TP53, MYM88, and PDGFB between dataset1 and dataset2 are different, despite the fact that distributions of case and control for those genes in each dataset are the same, suggesting that distributions in different datasets should be different (**Table S3**). Furthermore, the previously assumed distributions and variety of observed distributions belong to the Pearson distribution family. Hence, Pearson distribution family will be a necessary assumption for the distribution in microarray data. Any distribution can be characterized by a number of moments and the moments of a distribution describe the nature of its distribution [24]. *SWang* can use existing moments to detect DEGs via determining whether the distributions of case and control are the same. Under such circumstance, the general *SWang* test will be better for detecting DEGs.

In conclusion, *SWang* has significant performance with unbiased estimation of skewness and kurtosis under small sample sizes, and is a method to test the differences of the distributions between case and control for complex distribution of microarray data. Thus it can detect DEGs with low FPR and high SP when applied in microarray data analysis comparing to the other four methods.

As the microarray technologies have been widely used during the past decade, enormous data have been accumulated. How to extract the meaningful biological information from them is still a challenge. Our new method provides a new alternative and powerful way to recognize the DEGs. It is expected that revisiting the microarray data with our method could lead to the discovery of new biological knowledge and new insight into mechanisms for old biological processes and diseases.

## Materials and Methods

### Datasets

Two different datasets of breast cancer were used. The first dataset (Dataset1), used in the development of the *q* value method, was downloaded at http://research.nhgri.nih.gov/microarray/NEJMSupplement [28]. It consists of 3,226 genes on sample size n1 = 7 of BRCA1 arrays and sample size n2 = 8 of BRCA2 arrays. The second dataset (Dataset2) was downloaded from GEO (GES8193) and is an expression dataset from age-dichotomized ER+ breast tumors. We followed the original experimental design and divided the Dataset2 into two groups: one is used as control with the age ≤45; the other is as case with the age ≥70.

### Semiquantitative RT-PCR analysis

Total RNA was extracted with Trizol Reagent (Invitrogen) based on American Type Culture Collection's instructions, from which all breast cancer cell lines were obtained. Then cDNA was synthesized from total RNA using PrimeScriptTM RT reagent Kit (TaKaRa). The PCR reaction to amplify DNA fragments was performed at 94°C for 30 seconds, 55°C for 25 cycles of 30 seconds each, and 72°C for seconds.

### *SWang* methods

Assume the expression values of genes from *m* samples of control group are 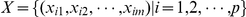, where the *p* is total size of genes, and the expression values of genes from *n* samples of case group are 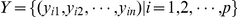. Also, the raw data transformed with logarithm base 2 are assumed normal distribution.

Previous statistical methods, such as T-test and its derivations, ANOVA and its derivations, and Fold-change, do not consider information of means, variances, skewness, and kurtosis simultaneously. We can determine whether a gene is differentially expressed solely based on the mean difference. However, it will be difficult for us to determine the differential expression of genes if the paired means of the gene expression levels between the control and case have no difference. Under such circumstance, we need to consider more information besides the means, such as variances, skewness, and kurtosis. Here, the null hypothesis is that the mean, variance, skewness and kurtosis between case group and control group are equal.

Accordingly, 
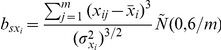
 is the skewness of 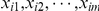, which is appropriated to normal distribution with mean as 0 and variance 6/m. 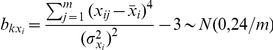 is the kurtosis of 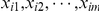, which is appropriated to normal distribution (mean = 0, variance = 24/m) [22], [45]. Similarly, 
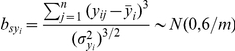
 is the skewness of 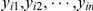, which is appropriated to normal distribution (mean = 0, variance = 6/n). 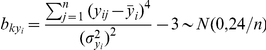 is the kurtosis of 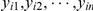, which is appropriated to normal distribution with mean as 0 and variance as 24/n. It can be proven that the kurtosis and skewness are independent of the mean and variance (**File S1**). Therefore, it is necessary to consider the skewness and kurtosis simultaneously when applying statistical methods to select the DEGs.

In real biological system, many regulatory mechanisms, like positive and negative feedback loops have great impact on the gene expression levels. The effect of perturbing the expression of any one gene will most likely lead to a cascade throughout the transcriptional regulatory network, affecting the expression of many other genes. Subsequently, changes of other genes in expression would conversely have the effect on the expression level of the perturbed gene due to the potential feedback control mechanism [46], [47]. Eventually, the feedback regulatory loops will make the perturbation of those genes convergent, and the microarray data is actually a snapshot that catches the homeostasis of an organism or cells at such specific time point; this leads to the microarray data not being the index of the initial differential expression for the given set of genes. Instead, it reflects the consequence of interactions among the genes which is composed of their initial expression level, the fluctuation of their expression, and the interaction among the functional related genes. We can construct a nonlinear gene interaction model for this scenario as follows,
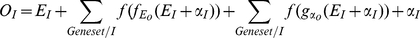
(5)where *I* represents the initial perturbed gene(s) in a biological system under experimental condition, 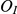 is the observed or measured expression value of the *I* gene(s) on microarray, 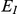 means the initial expression value of the *I* gene(s), 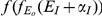 indicates the function of the *I* gene(s) expression caused by expression of other genes that have been affected by the expression and fluctuation of the *I* gene(s), 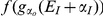 refers to the function of the *I* gene(s) expression caused by the fluctuation of other genes that have been affected by expression and fluctuation of the *I* gene(s), 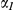 is the fluctuation of the *I* gene(s) and follows a normal distribution, 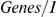 means the complementary set of *I*, which is also a set of all genes except *I*.

For normal distribution whose kernel is 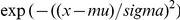, we assume that 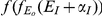 and 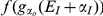 are nonlinear function because gene expression regulations are non-linear [48]. For simplicity, we can use quadratic function to construct a model for gene interactions. The function (11) in **File S1** indicates that from the biology view, it is not sufficient to only consider the differences between means or variances during DEGs detection.

When mean, variance, skewness, and kurtosis of gene expressions are the same, the genes can be regarded as not differentially expressed. We transform the estimation of central moments to raw moments through the functions 6–10 (**File S1**). The raw moments could be unbiasedly estimated by mapping to their corresponding to sample raw moments for any sample sizes greater than 4 [22]. Hence, the null hypothesis is that the mean, the average of squares, third moment, and fourth moment of gene expression between case group and control group are equal.

Based on the hypothesis, we can deduce a new null hypothesis that the one to four raw moment(s) of both the case and the control are equal which means H_0_: 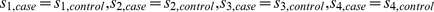 (**File S1**). Here, 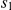, 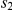, 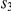, and 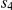 are raw moments. The first four raw moments of any sampling distribution can be separately estimated in an unbiased manner but all of them can not take the expected values simultaneously [22]. For central-moments like mean, variance, skewness and kurtosis, and raw moments, the sample size should be greater than 4 when estimating the first four central and raw moments unbiasedly [22]. In the Pearson distribution family, a reliable estimator of kurtosis can be obtained at relatively small sample sizes [24]. Under the derived null hypothesis, we can construct the *SWang* test which can be proven to appropriately follow F distribution whose one freedom is *k* and the other is *n+m-k-1*. The *k* is the k^th^ raw moments and *n* is the sample size in case group while *m* is the sample size in control group. First, we let some notions on the *SWang* test as:
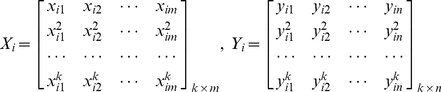
(7)
*X_i_* is the matrix of 1st-*k*th power of a gene expression in case group, *Y_i_* is the matrix of 1st-kth power of its expression in control group, 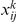 is a *k* power of the *i* gene expression value that is transformed using the base 2 logarithm for control, *x* is the gene expression of case group, *j* represents the sample. Similarly, 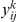 is a *k* power of the *i* gene expression value that is transformed using the base 2 logarithm for case, *y* gene expression of control group, *j* represents the sample.

According to the Statistics and Matrix theory, the mean of 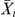 and 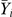 can be inferred as:
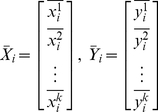
(8)Where 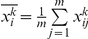 and 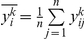. The deviation matrix D_1_ and D_2_ of the samples can be calculated as:
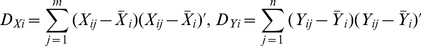
(9)Where 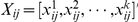, 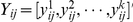.

According to the multivariate test [49], [50], [51], [52] which is a multivariate mean vector test while the 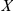 are drawn from multinormal distribution whose mean is 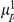 and *p*×*p* covariance matrix is 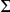 that is unknown, 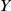 are drawn from multinormal distribution whose mean is 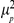 and covariance matrix is also 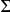. The multivariate test can be presented as:
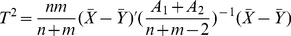
(10)where 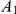 and 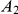 are the deviation matrix.

Like the multivariate test, the *SWang* test is,
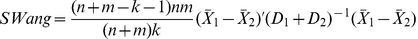
(11)
*SWang* test can be transformed to be appropriate to F-distribution with two freedom *k* and *n+m-k-1*(Proof in **File S2**).

However it is not always clear whether the matrix is nonsingular or not, so we utilize the generalized inverse of matrix rather than the inverse of matrix [11] to calculate *SWang*.
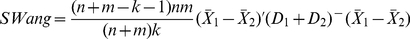
(12)Since there exists different permutation for different moments, the *SWang* can also be signified as *SWang*(*h,k*), where *h* is the lowest moment and *k* is the highest moment. *SWang*(h,k) utilizes the information from *h* moment to *k* moment, such as *SWang*(1,3) use the information from the first moment to third moment. Since the selection of the moments depends on distribution and sample size, we recommend *h* to be 1 and *k* to be 4. However, if the *k* is equal to 1, *SWang*(1,1) is a square of T-test (**File S3**), else if *k* is greater than 2, the test is not a general test. Our statistical package is available upon requested.

According to the theorem 2.3.11 [24], when *F_X_(x)* and *F_Y_(y)* are two cumulative distribution functions in which all moments exist, and *X* and *Y* have bounded support, then *F_X_(u)* equals to *F_Y_(u)* for all *u* if and only if *E(X^r^)* = *E(Y^r^)* for all integers r = 0, 1, 2,…. Besides, the distribution of microarrays is assumed to be of the Pearson distribution family whose highest moment is kurtosis. Since *SWang* incorporate the first four moments, *SWang* can be used to test whether the distributions of case and control are the same and detect DEGs.

## Supporting Information

File S1 Formula of T-test and F-test, proof of independence between skewness, kurtosis, mean, and variance, transform of moments, biological model
(0.23 MB DOC)

File S2 Proof of SWang test
(0.06 MB DOC)

File S3 General inverse of matrix,relation between SWang test and T-test
(0.05 MB DOC)

File S4 The pseudo codes of simulation on SWang test, T-test, F-test, SAM, Fold-change to calculate false positive rate and statistics power, and the SAS/iml code for calculate SWang
(0.05 MB DOC)

File S5 This file contains supporting information tables and a list of reference
(0.18 MB DOC)

Figure S1 False positive rate of T-test, F-test, Fold-change, SAM(0.3), and SWang respectively, with 5 Case-control Samples from Uniform distribution. Under Complex distribution for case and control. A: False positive rate of SWang(1,2) and other methods. B: False positive rate of SWang(1,3) and other methods. C: False positive rate of SWang(1,4) and other methods. D: False positive rate of SWang(2,3) and other methods. E: False positive rate of SWang(2,4) and other methods. F: False positive rate of SWang(1,3) and other methods. The false positive rate of T-test(black spotline), F-test(gray spotline), Fold change(green spotline), SWang(blue spotline), and SAM(0.3)(red spotline) with cutoff of p-value.
(4.80 MB TIF)

Figure S2 False positive rate of T-test, F-test, Fold-change, SAM(0.3), and SWang, respectively, with 5 Case-control Samples from complex distribution, respectively. Under Normal distribution for case and control. A: False positive rate of SWang(1,2) and other methods. B: False positive rate of SWang(1,3) and other methods. C: False positive rate of SWang(1,4) and other methods. D: False positive rate of SWang(2,3) and other methods. E: False positive rate of SWang(2,4) and other methods. F: False positive rate of SWang(1,3) and other methods. The false positive rate of T-test(black spotline), F-test(gray spotline), Fold change(green spotline), SWang(blue spotline), and SAM(0.3)(red spotline) with cutoff of p-value.
(4.80 MB TIF)

Figure S3 False positive rate of T-test, F-test, Fold-change, SAM(0.3), and SWang, respectively, with different Samples from Uniform distribution. Under Uniform distribution for case and control. A: False positive rate of SWang(1,2) and other methods. B: False positive rate of SWang(1,3) and other methods. C: False positive rate of SWang(1,4) and other methods. D: False positive rate of SWang(2,3) and other methods. E: False positive rate of SWang(2,4) and other methods. F: False positive rate of SWang(1,3) and other methods. SS = 3, 6, 8, 12, 20, 40, 100, and 200 mean that there exist 3, 6, 8, 12, 20, 40, 100, and 200 case-control samples, respectively. The false positive rate of T-test(black spotline), F-test(gray spotline), Fold change(green spotline), SWang(blue spotline), and SAM(0.3)(red spotline) with cutoff of p-value. (Note: FPR of SAM and Fold-change for large sample sizes are similar to those of SAM and Fold-change for 3 samples. To better display the FPR of methods, the graphs will not plot the FPR of SAM and Fold-change when the sample size is greater than 3.)
(9.06 MB TIF)

Figure S4 False positive rate of T-test, F-test, Fold-change, SAM(0.3), and SWang, respectively, with different Samples from Normal distribution. Under Normal distribution for case and control. A: False positive rate of SWang(1,2) and other methods. B: False positive rate of SWang(1,3) and other methods. C: False positive rate of SWang(1,4) and other methods. D: False positive rate of SWang(2,3) and other methods. E: False positive rate of SWang(2,4) and other methods. F: False positive rate of SWang(1,3) and other methods. SS = 3, 6, 8, 12, 20, 40, 100, and 200 mean that there exist 3, 6, 8, 12, 20, 40, 100, and 200 case-control samples, respectively. The false positive rate of T-test(black spotline), F-test(gray spotline), Fold change(green spotline), SWang(blue spotline), and SAM(0.3)(red spotline) with cutoff of p-value.
(9.06 MB TIF)

Figure S5 False positive rate of T-test, F-test, Fold-change, SAM(0.3), and SWang, respectively, with different Samples from Complex distribution. Under complex distribution for case and control. A: False positive rate of SWang(1,2) and other methods. B: False positive rate of SWang(1,3) and other methods. C: False positive rate of SWang(1,4) and other methods. D: False positive rate of SWang(2,3) and other methods. E: False positive rate of SWang(2,4) and other methods. F: False positive rate of SWang(1,3) and other methods. SS = 3, 6, 8, 12, 20, 40, 100, and 200 mean that there exist 3, 6, 8, 12, 20, 40, 100, and 200 case-control samples, respectively. The false positive rate of T-test(black spotline), F-test(gray spotline), Fold change(green spotline), SWang(blue spotline), and SAM(0.3)(red spotline) with cutoff of p-value.
(9.06 MB TIF)

Figure S6 False positive rate of T-test, F-test, Fold-change, SAM(0.3), and SWang, respectively, with different Samples from Exponential distribution. Under Exponential distribution for case and control. A: False positive rate of SWang(1,2) and other methods. B: False positive rate of SWang(1,3) and other methods. C: False positive rate of SWang(1,4) and other methods. D: False positive rate of SWang(2,3) and other methods. E: False positive rate of SWang(2,4) and other methods. F: False positive rate of SWang(1,3) and other methods. SS = 3, 6, 8, 12, 20, 40, 100, and 200 mean that there exist 3, 6, 8, 12, 20, 40, 100, and 200 case-control samples, respectively. The false positive rate of T-test(black spotline), F-test(gray spotline), Fold change(green spotline), SWang(blue spotline), and SAM(0.3)(red spotline) with cutoff of p-value.
(9.06 MB TIF)

Figure S7 False positive rate of T-test, F-test, Fold-change, SAM(0.3), and SWang, respectively, with different Samples from Cauchy distribution. Under Cauchy distribution for case and control. A: False positive rate of SWang(1,2) and other methods. B: False positive rate of SWang(1,3) and other methods. C: False positive rate of SWang(1,4) and other methods. D: False positive rate of SWang(2,3) and other methods. E: False positive rate of SWang(2,4) and other methods. F: False positive rate of SWang(1,3) and other methods. SS = 3, 6, 8, 12, 20, 40, 100, and 200 mean that there exist 3, 6, 8, 12, 20, 40, 100, and 200 case-control samples, respectively. The false positive rate of T-test(black spotline), F-test(gray spotline), Fold change(green spotline), SWang(blue spotline), and SAM(0.3)(red spotline) with cutoff of p-value.
(9.06 MB TIF)

Figure S8 False positive rate of T-test, F-test, Fold-change, SAM(0.3) in ‘Spike-in’ dataset. A: False positive rate of SWang(1,2) and other methods. B: False positive rate of SWang(1,3) and other methods. C: False positive rate of SWang(1,4) and other methods. D: False positive rate of SWang(2,3) and other methods. E: False positive rate of SWang(2,4) and other methods. F: False positive rate of SWang(1,3) and other methods. The false positive rate of T-test(black spotline), F-test(grey spotline), Fold change(yellow spotline), SWang(blue spotline), and SAM(0.3)(red spotline) with cutoff of p-value.
(6.41 MB TIF)

Figure S9 Statistical power of T-test, F-test, Fold change, SAM(0.3), and SWang(1,4) with small sample sizes. A: Statistics power of those methods are under Normal distribution for case. B: under Exponetial distribution for case. C: under Uniform distribution for case. D: under Gamma distribution for case. E: under mixture of gamma and normal distribution of case group. F: under complex distribution which is a combination of various distribution for case.The statistical power of T-test (black spotline), F-test(gray spotline), Fold change(green spotline), SWang(blue spotline), and SAM(0.3)(red spotline) under simulation. (Note: the Size is equal to product of (Total-2)*m+n-3*Total+4, the total is the largest sample size is simulation.)
(9.06 MB TIF)

Figure S10 Statistical power of T-test, F-test, Fold change, SWang (1, 4), SAM(0.3) on Normal distribution and Normal distribution. The statistical power of T-test (black spotline), F-test(gray spotline), Fold change(green spotline), SWang(blue spotline), and SAM(0.3)(red spotline) under simulation. A: SWang(1,2), T-test, F-test, SAM(0.3) and Fold change. B: SWang(1,3), T-test, F-test, SAM(0.3) and Fold change. C: SWang(1,4), T-test, F-test, SAM(0.3) and Fold change; D: SWang(2,3), T-test, F-test, SAM(0.3) and Fold change. E: SWang(2,4), T-test, F-test, SAM(0.3) and Fold change. F: SWang(3, 4), T-test, F-test, SAM(0.3) and Fold change. (Note: the Size is equal to product of (Total-2)*m+n-3*Total+4, the total is the largest sample size is simulation.)
(9.06 MB TIF)

Figure S11 Statistical power of T-test, F-test, Fold change, SAM(0.3), and SWang based on Normal distribution and Normal distribution with small sample size. The statistical power of T-test (black spotline), F-test(gray spotline), Fold change(green spotline), SWang(blue spotline), and SAM(0.3)(red spotline) under simulation. A: SWang(1,2), T-test, F-test, SAM(0.3) and Fold change. B: SWang(1,3), T-test, F-test, SAM(0.3) and Fold change. C: SWang(1,4), T-test, F-test, SAM(0.3) and Fold change; D: SWang(2,3), T-test, F-test, SAM(0.3) and Fold change. E: SWang(2,4), T-test, F-test, SAM(0.3) and Fold change. F: SWang(3, 4), T-test, F-test, SAM(0.3) and Fold change. (Note: the Size is equal to the product of (Total-2)*m+n-3*Total+4, the total is the largest sample size is simulation.)
(9.06 MB TIF)

Figure S12 Statistical power of T-test, F-test, Fold-change, SWang (1, 4), SAM(0.3) on Exponential distribution and Normal distribution. The statistical power of T-test (black spotline), F-test(gray spotline), Fold change(green spotline), SWang(blue spotline), and SAM(0.3)(red spotline) under simulation. A: SWang(1,2), T-test, F-test, SAM(0.3) and Fold change. B: SWang(1,3), T-test, F-test, SAM(0.3) and Fold change. C: SWang(1,4), T-test, F-test, SAM(0.3) and Fold change; D: SWang(2,3), T-test, F-test, SAM(0.3) and Fold change. E: SWang(2,4), T-test, F-test, SAM(0.3) and Fold change. F: SWang(3, 4), T-test, F-test, SAM(0.3) and Fold-change. (Note: the Size is equal to the product of (Total-2)*m+n-3*Total+4, the total is the largest sample size is simulation.)
(9.06 MB TIF)

Figure S13 Statistical power of T-test, F-test, Fold change, SWang (1, 4), SAM(0.3) under Uniform distribution and Normal distribution. The statistical power of T-test (black spotline), F-test(gray spotline), Fold change(green spotline), SWang(blue spotline), and SAM(0.3)(red spotline) under simulation. A: SWang(1,2), T-test, F-test, SAM(0.3) and Fold change. B: SWang(1,3), T-test, F-test, SAM(0.3) and Fold change. C: SWang(1,4), T-test, F-test, SAM(0.3) and Fold change; D: SWang(2,3), T-test, F-test, SAM(0.3) and Fold change. E: SWang(2,4), T-test, F-test, SAM(0.3) and Fold change. F: SWang(3, 4), T-test, F-test, SAM(0.3) and Fold change. From figures, it shows that when the distribution of the case group's gene expression is complex distributions which is simple addition of Normal, Uniform, and Triangual distribution, the statistics power of SWang are decentralized. (Note: the Size is equal to the product of (Total-2)*m+n-3*Total+4, the total is the largest sample size is simulation.)
(9.06 MB TIF)

Figure S14 Statistical power of T-test, F-test, Fold change, SWang (1, 4), SAM(0.3) under Gamm distribution and Normal distribution. The statistical power of T-test (black spotline), F-test(gray spotline), Fold change(green spotline), SWang(blue spotline), and SAM(0.3)(red spotline) under simulation. A: SWang(1,2), T-test, F-test, SAM(0.3) and Fold change. B: SWang(1,3), T-test, F-test, SAM(0.3) and Fold change. C: SWang(1,4), T-test, F-test, SAM(0.3) and Fold change; D: SWang(2,3), T-test, F-test, SAM(0.3) and Fold change. E: SWang(2,4), T-test, F-test, SAM(0.3) and Fold change. F: SWang(3, 4), T-test, F-test, SAM(0.3) and Fold change. (Note: the Size is equal to the product of (Total-2)*m+n-3*Total+4, the total is the largest sample size is simulation.)
(9.06 MB TIF)

Figure S15 Statistical power of T-test, F-test, Fold change, SWang (1, 4), SAM(0.3) under mixture of Gamm & Normal distribution and Normal distribution. The statistical power of T-test (black spotline), F-test(gray spotline), Fold change(green spotline), SWang(blue spotline), and SAM(0.3)(red spotline) under simulation. A: SWang(1,2), T-test, F-test, SAM(0.3) and Fold change. B: SWang(1,3), T-test, F-test, SAM(0.3) and Fold change. C: SWang(1,4), T-test, F-test, SAM(0.3) and Fold change; D: SWang(2,3), T-test, F-test, SAM(0.3) and Fold change. E: SWang(2,4), T-test, F-test, SAM(0.3) and Fold change. F: SWang(3, 4), T-test, F-test, SAM(0.3) and Fold change. (Note: the Size is equal to the product of (Total-2)*m+n-3*Total+4, the total is the largest sample size is simulation.)
(9.06 MB TIF)

Figure S16 Statistical power of T-test, F-test, Fold change, SWang (1, 4), SAM(0.3) under complex distribution and Normal distribution. The statistical power of T-test (black spotline), F-test(gray spotline), Fold change(green spotline), SWang(blue spotline), and SAM(0.3)(red spotline) under simulation. A: SWang(1,2), T-test, F-test, SAM(0.3) and Fold change. B: SWang(1,3), T-test, F-test, SAM(0.3) and Fold change. C: SWang(1,4), T-test, F-test, SAM(0.3) and Fold change; D: SWang(2,3), T-test, F-test, SAM(0.3) and Fold change. E: SWang(2,4), T-test, F-test, SAM(0.3) and Fold change. F: SWang(3, 4), T-test, F-test, SAM(0.3) and Fold change. (Note: the Size is equal to the product of (Total-2)*m+n-3*Total+4, the total is the largest sample size is simulation.)
(9.06 MB TIF)

Figure S17 WNT Signal Pathway. The genes detected by SWang test but not by T-test, F-test, Fold-change, and SAM in Wnt signaling pathway based on KEGG. The genes in pink are the genes selected with our method.
(6.98 MB TIF)

Figure S18 5-venn diagram in dataset1. The cut-off of p-value of T-test, F-test, SAM(0.3), and SWang is 0.05, the cut-off of Fold-change is 2.
(8.71 MB TIF)

Figure S19 5-venn diagram in dataset2. The cut-off of p-value of T-test, F-test, SAM (0.3), and SWang is 0.05, while the cut-off of Fold-change is 2.
(8.75 MB TIF)

Figure S20 Semiquantative RT-PCR comparision. The genes which could not be detected by Swang test but were by the others are randomly selected. MCF-10A cells were cultured in DMEM/F12 with 10%FBS, 20ng/ml EGF, 0.5ug/ml Hydrocortisone, 0.01ug/ml Insulin and 0.1ug/ml Cholera toxin. MCF-7, SK-BR-3, MDA-MB-453 and MDA-MB-231 were maintained in DMEM with 10%FBS. PCR products were run on 2% agarose gel and then stained with ethidium bromide. Stained bands were visualized under UV light and photographed. The beta-actin used as an internal control.
(1.90 MB TIF)
