# Supplementary material for: Systematical Detection of Significant Genes in Microarray Data by Incorporating Gene Interaction Relationship in Biological Systems
Source: PLoS One. 2010 Oct 29;5(10):e13721. doi: 10.1371/journal.pone.0013721 (PMC2966410; doi:10.1371/journal.pone.0013721)
Supplement: File S1 — Formula of T-test and F-test, proof of independence between skewness, kurtosis, mean, and variance, transform of moments, biological model (0.23 MB DOC) [file pone.0013721.s001.doc]

S1 text

**T-test and F-test**

The null hypothesis of T-test is that $\bar{x}$ equals $\bar{y}$ while both of variances $\sigma_{x}^{2}$and$\sigma_{y}^{2}$ are known,

$t=\frac{\bar{x}-\bar{y}}{\sqrt{\frac{\sigma_{x}^{2}}{n}+\frac{\sigma_{y}^{2}}{m}}}$ （1）

The $\bar{x}_{i}=\sum_{j=1}^{m} x_{ij}/m$is the mean of $x_{i1},x_{i2},\cdots,x_{im}$, the $\bar{y}_{i}=\sum_{j=1}^{n} y_{ij}/n$is mean of $y_{i1},y_{i2},\cdots,y_{in}$.

However, since both of the variances, $\sigma_{x}^{2}$and$\sigma_{y}^{2}$, are unknown, the T-test should be

$t=\frac{\bar{x}_{i}-\bar{y}_{i}}{\sqrt{\frac{s_{x_{i}}^{2}}{n}+\frac{s_{y_{i}}^{2}}{m}}}$ （2）

Therefore,$s_{x_{i}}^{2}=\sum_{j=1}^{m} (x_{ij}-\bar{x}_{i})^{2}/(m-1)$is the variance of $x_{i1},x_{i2},\cdots,x_{im}$, $s_{y_{j}}^{2}=\sum_{j=1}^{n} (y_{ij}-\bar{y}_{i})^{2}/(n-1)$is the variance of $y_{i1},y_{i2},\cdots,y_{in}$ and the degree of freedom for T-test should be

$l=\frac{\left(s_{x_{i}}^{2}/n+s_{y_{i}}^{2}/m\right)^{2}}{(\left(s_{x_{i}}^{2}/n\right)^{2}/(n-1)+\left(s_{y_{i}}^{2}/m\right)^{2}/(m-1))}$ (3)

The null hypothesis of F-test is that$\sigma_{x}^{2}$ is equal to$\sigma_{y}^{2}$, then

$F=\frac{s_{x_{i}}^{2}}{s_{y_{i}}^{2}}$ (4)

where the degrees of freedom of F distribution are *m-1* and *n-1*. Many methods can test the variances’ equality. Analysis of variance (ANOVA) will also be an F-test.

**The proof of independence between skewness, kurtosis and mean with variance.**

Given $x_{i1},x_{i2},\cdots,x_{im}$ drawn from normal distribution with its mean as *mu* and standard deviance as *sigma*, we normalize the samples with the (x_ij_-*mu*)/*sigma*, but keep skewness and kurtosis unchanged. Hence, both skewness and kurtosis are independent to normal distribution parameters *mu* and *sigma*. However, $\bar{x}_{i}$ is the estimation of *mu* and $\sigma_{x_{i}}^{2}$ is the estimation of square of *sigma*. Finally, it can be proved that skewness and kurtosis are independent to $\bar{x}_{i}$ and$\sigma_{x_{i}}^{2}$. And the mean and variance are independent, hence we can use vectors whose diagonal are different moments.

**Transformation of central moments with non-central moments**

$m_{r}=\frac{1}{n}\sum_{i=1}^{n} (x_{i}-\bar{x})^{r}$ (5)

$m_{1}=0$ (6)

$m_{2}=-\frac{S_{1}^{2}}{n^{2}}+\frac{S_{2}}{n}$ (7)

$m_{3}=\frac{6S_{1}^{3}}{n^{3}}-\frac{3S_{1}S_{3}}{n^{2}}+\frac{S_{3}}{n}$ (8)

$m_{4}=-\frac{3S_{1}^{4}}{n^{4}}+\frac{6S_{1}^{2}S_{2}}{n^{3}}-\frac{4S_{1}S_{3}}{n^{2}}+\frac{S_{4}}{n}$ (9)

$m_{5}=\frac{4S_{1}^{5}}{n^{5}}-\frac{10S_{1}^{3}S_{2}}{n^{4}}+\frac{10S_{1}^{2}S_{3}}{n^{3}}-\frac{10S_{1}S_{4}}{n^{2}}+\frac{S_{5}}{n}$ (10)

Where $S_{r}=\frac{1}{n}\sum_{i=1}^{n} x_{i}^{r}$, and $r=1,2,3,\cdots,k$(k≥2) and $m_{1}$, $m_{2}$,$m_{3}$,$m_{4}$, and $m_{5}$ are central moments [1]. In biological system, the maximum moments should be four as proved below. Hence, the null hypothesis can transform into that the $S_{1}$, $S_{2}$, $S_{3}$ and $S_{4}$ are equal. According to Cramer theory [2], if X is normal distribution with mean vector $\mu$and covariance matrix $\Sigma$, then where $g$is a mapping $g: R^{d}\mapsto R^{k}$such that is continuous in a neighborhood of $\mu\in R^{d}$. $S_{1}\sim N(\mu, \frac{\sigma^{2}}{n})$, $S_{2}\sim N(\mu^{2}, \frac{4\mu^{2}\sigma^{2}}{n})$, $S_{3}\sim N(\mu^{3}, \frac{9\mu^{4}\sigma^{2}}{n})$, $S_{4}\sim N(\mu^{4}, \frac{16\mu^{6}\sigma^{2}}{n})$.

According to Cramer theory [2], assuming finite eighth moments, from central limit law,

**Non-linear Biological model for genes interaction (NGIM)**

We can obtain function (11) as follows:

$\begin{matrix} O_{I}=E_{I}+\sum_{Geneset/I} f(f_{E_{o}}(E_{I}+\alpha_{I}))+\sum_{Geneset/I} f(g_{\alpha_{o}}(E_{I}+\alpha_{I}))+\alpha_{I} \\ =E_{I}+\sum_{Geneset/} w_{I}(w_{o}(E_{I}+\alpha_{I})^{2})^{2}+\sum_{Geneset/I} w_{I}(\alpha_{o}(E_{I}+\alpha_{I})^{2})+\alpha_{I} \\ =E_{I}+\sum_{Geneset/I} w_{I}w_{o}^{2}(E_{I}+\alpha_{InGeVa})^{4}+\sum_{Geneset/I} w_{I}(\alpha_{o}(E_{I}+\alpha_{I})^{2})+\alpha_{I} \\ =E_{I}+\sum_{Geneset/I} w_{I}w_{o}^{2}(E_{I}^{4}+4E_{I}^{3}\alpha_{I}+6E_{I}^{2}\alpha_{I}^{2}+4E_{I}\alpha_{I}^{3}+\alpha_{I}^{4})+ \\ \sum_{Geneset/I} w_{I}(\alpha_{o}(E_{I}+\alpha_{I})^{2})+\alpha_{I} \end{matrix}$ (11)

where *I* represents the initial expressed gene(s) in a biological system under experimental condition, $O_{I}$is the observed or measured expression value of the *I* gene(s) on microarray, $E_{I}$means the initial expression value of the *I* gene(s), $f(f_{E_{o}}(E_{I}+\alpha_{I}))$ indicates the function of the *I* gene(s) expression caused by expression of other genes that have been affected by the expression and fluctuation of the *I* gene(s), $f(g_{\alpha_{o}}(E_{I}+\alpha_{I}))$ refers to the function of the *I* gene(s) expression caused by the fluctuation of other genes that have been affected by expression and fluctuation of the *I* gene(s), $\alpha_{I}$is the fluctuation of the *I* gene(s) and follows a normal distribution. For normal distribution whose kernel is $\exp(-((x-mu)/sigma)^{2})$, we assume that $f(f_{E_{o}}(E_{I}+\alpha_{I}))$ and $f(g_{\alpha_{o}}(E_{I}+\alpha_{I}))$ are nonlinear function because gene expression regulations are non-linear [4], $Geneset/I$means the complementary set of *I*, which is also a set of all genes without *I*.$w_{I}$ represents the parameters of the expression value of the *I* gene(s) which other genes’ variation lead to change, $w_{o}$ denotes the parameters of the expression value of other genes which the *I* gene(s)causes. $E_{I}$demonstrates that the method should consider the difference of means, $E_{I}^{2}$indicates that the method should contain information of second moment, $E_{I}^{3}$ signify that the methods should comprise the third moment information, and $E_{I}^{4}$ means that the methods should contain the forth moment information. $\alpha_{I}$ means that the noise of gene expression could follow normal distribution, $\alpha_{I}^{2}$ indicates that the square of gene expression noise is under chi-square distribution. $\alpha_{I}^{3}$ refers that the cubic of gene expression noise belongs to complex distribution. $\alpha_{I}^{4}$ signifies that the quartic of gene expression noise fit complex distribution.

To exactly estimate the $E_{I}$,$E_{I}^{2}$,$E_{I}^{3}$,$E_{I}^{4}$, model(11) should be as a nonlinear structure equation model (NSEM), there are several methods to estimate NSEM [4,5]. In theory, to unbiasedly estimate NSEM, it is required that the sample size is at least greater than twice of the number of genes in Microarray. Practically, the sample size is insufficient to estimate NSEM. An alternative way to unbiasedly estimate NSEM is to eliminate the undifferentially expressed genes and to detect DEGS. Here,

$E_{I}=\frac{1}{n}\sum O_{Ij}$,

$E_{I}^{2}=\frac{1}{n}\sum O_{Ij}^{2}$,

$E_{I}^{3}=\frac{1}{n}\sum O_{Ij}^{3}$,

$$E_{I}^{4}=\frac{1}{n}\sum O_{Ij}^{4}$$

This method has certain bias, however, those functions in the method can be used simultaneously to decrease the bias in a nonlinear system and to illustrate that skeness and kurtosis are important in biological system.

**Reference**

1. Lehmann EL (1999) The Elements of Large-Sample Theory: Springer. 79-170 p.

2. S.Ferguson T (1996) A Course in Large Sample Theory: Chapman & Hall. 44-50 p.

3. Timm NH (2001) Applied Multivariate Analysis: Springer. 79-170 p..

4. T. Amemiya (1974) The Nonlinear Two-Stage Least-Squares Estimator. Journal of Econometrics 5: 105-110.

5. T. Amemiya (1977) The Maximum Likelihood Estimator and the Nonlinear Three-Stage Least Squares Estimator in the General Nonlinear Simultaneous Equation Model. Econometrica 45(4): 955-966.
