# Supplementary material for: Systematical Detection of Significant Genes in Microarray Data by Incorporating Gene Interaction Relationship in Biological Systems
Source: PLoS One. 2010 Oct 29;5(10):e13721. doi: 10.1371/journal.pone.0013721 (PMC2966410; doi:10.1371/journal.pone.0013721)
Supplement: File S2 — Proof of SWang test (0.06 MB DOC) [file pone.0013721.s002.doc]

**S2 text**

**The proof of *SWang***

Under the null hypothesis, we can prove that the distribution of $\bar{X}-\bar{Y}$is normal distribution, which mean is zero vector and covariance matrix is

$(\frac{1}{n}+\frac{1}{m})\Sigma$ (12)

Therefore,

$\sqrt{\frac{nm}{m+n}}(\bar{X}-\bar{Y})$ (13)

is multi-normal distribution which mean is zero vector and covariance matrix is $\Sigma$.

$A_{1}=\sum_{j=1}^{n} (X_{(a)}-\bar{X})(X_{(a)}-\bar{X})'$ , $A_{2}=\sum_{a=1}^{m} (Y_{(a)}-\bar{Y})(Y_{(a)}-\bar{Y})'$ (14)

The *A1* and *A2* is Wishart distribution with the freedom *n-1* and the covariance matrix is still$\Sigma$.

Since one of the properties for Wishart distribution is addictive that different samples independently drawn from the same Wishart distribution can be added, and the sum will also appropriate to Whishart distribution [1].

$\sum W_{i}\sim W(n,\Sigma),W_{i}\sim W(n_{i},\Sigma),n=\sum n_{i}$ (15)

so it can prove

$A_{1}+A_{2}\sim W(n+m-2,\Sigma)$ (16)

then, we can obtain that $T^{2}$ is the Hotelling’s $T^{2}$ distribution with freedom *p* and *n+m-2*. Finally, according to the relationship between $T^{2}$ distribution and F distribution, we can transform the $T^{2}$ test into an F-test[2].

$F=\frac{(n+m-2)-p+1}{(n+m-2)*p}T^{2}\sim F(p,n+m-p-1)$ (17)

Therefore, *SWang* can be transformed approximately to be F-distribution.

$SWang=\frac{(n+m-k-1)nm}{(n+m)k}(\bar{X}_{1}-\bar{X}_{2})'(D_{1}+D_{2})^{-}(\bar{X}_{1}-\bar{X}_{2})\sim F(k,n+m-k-1)$ (18)

**Reference**

1. Timm NH (2001) Applied Multivariate Analysis: Springer. 79-170 p.

2. Härdle W, L S (2007) Applied Multivariate statistical Analysis: Springer. 39-196 p.
