# Supplementary material for: Systematical Detection of Significant Genes in Microarray Data by Incorporating Gene Interaction Relationship in Biological Systems
Source: PLoS One. 2010 Oct 29;5(10):e13721. doi: 10.1371/journal.pone.0013721 (PMC2966410; doi:10.1371/journal.pone.0013721)
Supplement: File S3 — General inverse of matrix,relation between SWang test and T-test (0.05 MB DOC) [file pone.0013721.s003.doc]

**S3 text**

**Determining which type of the general inverse can be used**

Characterizations of g-inverse

The customary notation for a g-inverse of A is A- .

1. For most cases, matrix A∈M_m×n_, A^-^ ∈M_m×n_ exists, but the inverse of A does not always exist if A is singular.
2. *SWang* and *SWang*(h,k)

$$\begin{matrix} SWang=\frac{(n+m-2)-(k-1+1)+1}{n+m-2}T^{2} \\ =\frac{(n+m-2)-(k-1+1)+1}{(n+m-2)*(k-1+1)}\frac{nm}{(n+m)}(\bar{X}_{1}-\bar{X}_{2})'(\frac{D_{1}+D_{2}}{n+m-2})^{-1}(\bar{X}_{1}-\bar{X}_{2}) \\ =\frac{(n+m-2)-(k-1+1)+1}{(n+m-2)*(k-1+1)}\frac{nm}{(n+m)}(n+m-2)(\bar{X}_{1}-\bar{X}_{2})'(D_{1}+D_{2})^{-1}(\bar{X}_{1}-\bar{X}_{2}) \\ =\frac{(n+m-k-1)nm}{(n+m)k}(\bar{X}_{1}-\bar{X}_{2})'(D_{1}+D_{2})^{-1}(\bar{X}_{1}-\bar{X}_{2}) \end{matrix}$$

$$\begin{matrix} SWang(h,k)=\frac{(n+m-2)-(k-h+1)+1}{n+m-2}T^{2} \\ =\frac{(n+m-2)-(k-h+1)+1}{(n+m-2)*(k-h+1)}\frac{nm}{(n+m)}(\bar{X}_{1}-\bar{X}_{2})'(\frac{D_{1}+D_{2}}{n+m-2})^{-1}(\bar{X}_{1}-\bar{X}_{2}) \\ =\frac{(n+m-2)-(k-h+1)+1}{(n+m-2)*(k-h+1)}\frac{nm}{(n+m)}(n+m-2)(\bar{X}_{1}-\bar{X}_{2})'(D_{1}+D_{2})^{-1}(\bar{X}_{1}-\bar{X}_{2}) \\ =\frac{(n+m-k+h-2)nm}{(n+m)*(k-h+1)}(\bar{X}_{1}-\bar{X}_{2})'(D_{1}+D_{2})^{-1}(\bar{X}_{1}-\bar{X}_{2}) \end{matrix}$$

If *k* = *h* =1, *SWang* is the square of T-test whose variances are unknown but equal.

$$\begin{matrix} SWang(h,k)=\frac{(n+m-2)-(1-1+1)+1}{n+m-2}T^{2} \\ =\frac{(n+m-2)-(1-1+1)+1}{(n+m-2)*(1-1+1)}\frac{nm}{(n+m)}(\bar{x}_{1}-\bar{x}_{2})'(\frac{d_{1}+d_{2}}{n+m-2})^{-1}(\bar{x}_{1}-\bar{x}_{2}) \\ =\frac{(n+m-2)-(1-1+1)+1}{(n+m-2)*(1-1+1)}\frac{nm}{(n+m)}(n+m-2)(\bar{x}_{1}-\bar{x}_{2})'(d_{1}+d_{2})^{-1}(\bar{x}_{1}-\bar{x}_{2}) \\ =\frac{(n+m-2)nm}{(n+m)*(1-1+1)}(\bar{x}_{1}-\bar{x}_{2})'(d_{1}+d_{2})^{-1}(\bar{x}_{1}-\bar{x}_{2}) \\ =\frac{(n+m-2)nm}{(n+m)}(\bar{x}_{1}-\bar{x}_{2})'(d_{1}+d_{2})^{-1}(\bar{x}_{1}-\bar{x}_{2}) \\ =(\frac{\bar{x}_{1}-\bar{x}_{2}}{\sqrt{\frac{nm}{m+n}}(\frac{d_{1}+d_{2}}{n+m-2})})^{2} \\ =(\frac{\bar{x}_{1}-\bar{x}_{2}}{\sqrt{\frac{nm}{m+n}}(\frac{(n-1)s_{1}+(m-1)s_{2}}{n+m-2})})^{2} \end{matrix}$$

Hence, we know *SWang*(1,1) is the square of T-test which is approximate to be F-distribution.
