# Supplementary material for: Systematical Detection of Significant Genes in Microarray Data by Incorporating Gene Interaction Relationship in Biological Systems
Source: PLoS One. 2010 Oct 29;5(10):e13721. doi: 10.1371/journal.pone.0013721 (PMC2966410; doi:10.1371/journal.pone.0013721)
Supplement: File S4 — The pseudo codes of simulation on SWang test, T-test, F-test, SAM, Fold-change to calculate false positive rate and statistics power, and the SAS/iml code for calculate SWang (0.05 MB DOC) [file pone.0013721.s004.doc]

**S4 text**

**Pseudo code for simulation to calculating false positive rate**

/* generating microarrays*/

For case_number in 3, 4, 5, 6, 8, 12, 20, 40, 100, and 200

For control_number in 3, 4, 5, 6, 8, 12, 20, 40, 100, and 200

1. Generating random number as case from special distribution
2. Generating random number as control from the same distribution of control

Repeat (a) (b) 10000 times /*generating genes*/

End

End

/* set real DEGs in the generated genes */

For loop in 1 to 10000

Generate random number from Binomial distribution with successful ration 0.2.

End

/*Detecting DEGs and Calculate false positve rate*/

For cutoff_p-value in 0 to 0.1 by 0.002

/*generating microarrays*/

Calculate SWang and p-value of SWang,

If p-value is less than , the “gene” is DEG.

Construct 2X2 crosstable to calculate false positive rate

End.

**Pseudo code for simulation to calculating Statistics power**

/* generating microarrays with gene interaction*/

For case_number in 3 to 53

For control_number in 3 to 53

1. Generating random number as case from distribution like Normal, Gamma, Exponential, Uniform, mixture of normal
2. Generating random number as control from normal
3. Current random number will be a variable for the quadratic function

Repeat (a) (b) (c) 10000 times

End

End

/*Detecting DEGs and Calculate Statistics power*/

Calculate SWang, count the p-value of SWang of “gene” is less than 0.05.

Calculate Statiscs power.

**The codes for SWang in SAS proc iml**

/* this programme is wroten by Junwei Wang*/

/* Dec 2008 */

/* the programme is used to simulate the microarray data*/

Proc iml;

start SWang(x,y);

n3=nrow(x); n1=ncol(x); n2=ncol(y);

do i=1 to n3;

do k=&k1 to &k2;

m1=x[i,]##k;

mm1=mm1//m1;

m2=y[i,]##k;

mm2=mm2//m2;

end;

mean1=mm1[,:];

nn1=repeat(mean1,1,n1);

s1=mm1-nn1;

var1=s1*s1`;

mean2=mm2[,:];

nn2=repeat(mean2,1,n2);

s2=mm2-nn2;

var2=s2*t(s2);

mean=mean1-mean2;

var=((n1-1)*var1+(n2-1)*var2)/(n1+n2-2);

xx=ginv(var);

value=abs(((n1+n2-1-(&k2-&k1+1))/(&k2-&k1+1))*(n1*n2)/(n1+n2)*mean`*xx*mean);

p=1-probf(value,&k2-&k1,n1+n2-(&k2-&k1)-1);

value1=value1//value;

pp=pp//p;

free m1 m2 mm1 mm2 mean1 nn1 s1 var1 mean2 nn2 s2 var2;

end;

return(pp);

free n1 n2 n3;

finish;
