# Supplementary material for: Systematical Detection of Significant Genes in Microarray Data by Incorporating Gene Interaction Relationship in Biological Systems
Source: PLoS One. 2010 Oct 29;5(10):e13721. doi: 10.1371/journal.pone.0013721 (PMC2966410; doi:10.1371/journal.pone.0013721)
Supplement: File S5 — This file contains supporting information tables and a list of reference (0.18 MB DOC) [file pone.0013721.s005.doc]

**Table S1**. Results selected with *SWang*(1,4) from Dataset1 and Dataset2.

Genes that are in the same cancer related signaling pathways and not in the significant gene lists that T-test and q-value selected. Several genes confirmed by our experiment.

| **Gene** | **GeneID** | **Description** |
| --- | --- | --- |
| CCND1 | 595 | A strong correlation between CCND1 amplification and its protein expression in breast cancer [1]. |
| EGFR | 1956 | Metaplastic breast carcinomas frequently overexpressed EGFR, which was associated with EGFR gene amplification in one-third of cases [2]. However, EGFR gene amplification is an infrequent event in breast cancer, occurring in only 6% of tumor [3]. |
| IL1R1 | 3554 | IL1R1 levels are slightly modulated in BCAR1 cells and its microarry expression is well reproduced by Q-PCR [4] [5]. |
| MCM4 | 4173 | The upregulated MCM4 gene in our result is one of the genes involved in DNA replication and cell cycle, it has been reported that mutation in MCM play a role in cancer development in mice and may increase breast cancer risk in humans [6]. |
| MYD88 | 4615 | MyD88, an adaptor protein which is known to mediate the signaling of toll-like receptor (TLR), has been reported to mediate IFN-γ- induced MAP kinase activation and PD-L1 expression. And TLR is expressed in breast cancer [7]. It was shown that chemopreventive agents potentiate IFN-γ-induced PD-L1 expression in human breast cancer cells [8]. |
| PDGFB | 5155 | Overexpression of PDGF-B was associated with peripheral blood, sentinel lymph node and bone marrow micro-metastasis in breast cancer [9]. |
| RRAS2 | 22800 | The gene Rras2, also known as TC21, possibility over-expressed and contributed to aberrant growth properties of breast carcinoma cells [10]. |
| TP53 | 7157 | The TP53 gene (p53) is found altered in breast carcinomas in approximately 20–40% of all cases depending on tumor size and stage of the disease. It seems to be an early event in breast tumorigenesis [11]; TP53 mutation status and gene-expression based groups are important survival markers of breast cancer, and these molecular markers may provide prognostic information that complements clinical variables [12]; common variation in the TP53 gene could modify the risk of invasive breast cancer [13]. |
| FGFR1 | 2260 | FGFR1 has a role in preventing progression of breast neoplasms [14]. |
| FGFR2 | 2263 | FGFR2 and MAP3K1 are involved in breast cancer susceptibility and confer their effects primarily in ER+ and PR+ tumors [15]. In human breast cancer, expression of FGFR2 is estrogen receptor (ER)-dependent and correlates with a lower rate of apoptosis; genetic variants in FGFR2 may contribute to breast cancer occurrence in Chinese women [16]. |
| MAP2K2 | 5605 | MEK2 (MAP2K2) protein’s level was increased in breast carcinoma tissue compared with those in adjacent normal tissues [17]. |
| LIF | 3976 | LIF promoter is transcriptionally active in human breast cancer cells and its activity can be modulated by progestins and anti-progestins in cells expressing the LIF protein [18]. |
| PGF | 5228 | The expression of PGF is up-regulation in the clinical experiment Normal Fibroadenoma vs Invasive Lobular Carcinoma measured by cDNA Microarray [19]. |
| PML | 5371 | PML protein expression was reduced in breast cancer [20],[21]. |
| RASA1 | 5921 | The expression of RASA1 is up-regulated in clinical experiment BRCA1 mutation positive vs BRCA2 mutation positive, Sporadic measured by cDNA Microarray [22]. |
| RPS6KA6  (Rsk4) | 422273 | RT-PCR data show high expression of putative tumor suppressor genes Rsk4 and RbAp46 in 47% and 79% of breast carcinoma cases, respectively, whereas Cldn2 was down-regulated in 52% of breast cancer cases compared with normal adjacent tissues [23]. |
| SERPINB4  (SCCA2) | 6318 | SCCA2 regulates cell migration and invasion via E-cadherin expression, suggesting that SCCA2 may be involved in cancer behavior such as invasion or metastasis [24]. |
| WNT1 | 7471 | Wnt-1 expressed a high level in MCF-7 cell line [20]. |
| BCR | 613 | See Semiquantative-RT-PCR |
| CD8A | 925 | See Semiquantative-RT-PCR |
| CYB561 | 1534 | See Semiquantative-RT-PCR |
| DTX3 | 196403 | See Semiquantative-RT-PCR |
| FZD4 | 8322 | See Semiquantative-RT-PCR |
| GAS2L1 | 10634 | See Semiquantative-RT-PCR |
| IDS | 3423 | See Semiquantative-RT-PCR |
| NPTXR | 23467 | See Semiquantative-RT-PCR |
| NUP210 | 23225 | See Semiquantative-RT-PCR |
| SAR1A | 56681 | See Semiquantative-RT-PCR |
| UNC45A | 55898 | See Semiquantative-RT-PCR |

**Reference**

1. Somaia Elsheikh, Andrew R. Green, Mohammed A. Aleskandarany, Matthew Grainge, Claire E. Paish, et al. (2008) CCND1 amplification and cyclin D1 expression in breast cancer and their relation with proteomic subgroups and patient outcome Breast Cancer Research and Treatment 109: 325-335.
2. Bhargava R, Gerald WL, Li AR, Pan Q, Lal P, et al. (2005) EGFR gene amplification in breast cancer: correlation with epidermal growth factor receptor mRNA and protein expression and HER-2 status and absence of EGFR-activating mutations. Modern Pathology 18(8): 1027-1033.
3. Reis-Filho JS, Milanezi F, Carvalho S, Simpson PT, Steele D, et al. (2005) Metaplastic breast carcinomas exhibit EGFR, but not HER2, gene amplification and overexpression: immunohistochemical and chromogenic in situ hybridization analysis. Breast Cancer Research 7(6): R1028-1035.
4. Perou CM, Sørlie T, Eisen MB, van de Rijn M, Jeffrey SS, et al. (2000) Molecular portraits of human breast tumours. Nature 406(6797): 747-752..
5. Hofer EL, La Russa V, Honegger AE, Bullorsky EO, Bordenave RH, et al. (2005) Alteration on the expression of IL-1, PDGF, TGF-beta, EGF, and FGF receptors and c-Fos and c-Myc proteins in bone marrow mesenchymal stroma cells from advanced untreated lung and breast cancer patients. Stem Cells Dev 14: 587-594.
6. Gruvberger S, et al. (2001) Estrogen receptor status in breast cancer is associated with remarkably distinct gene expression patterns. Cancer Res 61:5979-5984.
7. Bo Huang, Jie Zhao, Hongxing Li, Kai-Li He, Yibang Chen, et al. (2005) Toll-Like Receptors on Tumor Cells Facilitate Evasion of Immune Surveillance. Cancer Research 65: 5009.
8. Zhang P, Su DM, Liang M FJ (2008) Chemopreventive agents induce programmed death-1-ligand 1(PD-L1) surface expression in breast cancer cells and promote PD-L1-mediated T cell apoptosis. Molecular Immunology 45: 1470-1476.
9. Zhu L, Loo WT, Cheng CW, LW. C (2006) Possible predictive markers related to micro-metastasis in breast cancer patients. Oncol Rep 15: 1217-1223.
10. Clark GJ, Kinch MS, Gilmer TM, Burridge K, and Der CJ (1996) Overexpression of the Ras-related TC21/R-Ras2 protein may contribute to the development of human breast cancers. Oncogene 12(1):169-76.
11. AL B-D (2003) TP53 and breast cancer. Hum Mutat 21: 292-300.
12. Langerød A, Zhao H, Borgan Ø, Nesland JM, Bukholm IR, et al. (2007) TP53 mutation status and gene expression profiles are powerful prognostic markers of breast cancer. Breast Cancer Research 9: R30.
13. Brian L. Sprague, Amy Trentham-Dietz, Montserrat Garcia-Closas, Polly A. Newcomb, Linda Titus-Ernstoff, et al. (2007) Genetic variation in TP53 and risk of breast cancer in a population-based case-control study. Carcinogenesis 28: 1680.
14. Jacquemier J, Sun ZZ, Penault-Llorca F, Geneix J, Devilard E, et al. (1998) FGF7 protein expression in human breast carcinomas. J Pathol 186: 269-274..
15. Elbauomy Elsheikh S, Green AR, Lambros MB, Turner NC, Grainge MJ, et al. (2007) FGFR1 amplification in breast carcinomas: a chromogenic in situ hybridisation analysis. Breast Cancer Res 9: R23.
16. Rebbeck TR, DeMichele A, Tran TV, Panossian S, Bunin GR, et al. (2009) Hormone-dependent effects of FGFR2 and MAP3K1 in breast cancer susceptibility in a population-based sample of post-menopausal African-American and European-American women. Carcinogenesis 30: 269-274..
17. Liang J, Chen P, Hu Z, Zhou X, Chen L, et al. (2008) Genetic variants in fibroblast growth factor receptor 2 (FGFR2) contribute to susceptibility of breast cancer in Chinese women. Carcinogenesis 29: 2341-2346.
18. Wang S, Zhu X, Zhang J, Qiao X, Ye Y, et al. (2002) [Significance of MEK-ERK cascade in the development of human breast carcinoma]. Zhonghua Wai Ke Za Zhi 40: 171-174.
19. Bamberger AM, Thuneke I, Schulte HM. (1998) Differential regulation of the human 'leukemia inhibitory factor' (LIF) promoter in T47D and MDA-MB 231 breast cancer cells. Breast Cancer Res Treat 47(2):153-161.
20. Perou CM, Sorlie T, Eisen MB, van de Rijn M, Jeffrey SS, et al. (2000) Molecular portraits of human breast tumours. Nature 406: 747-752.
21. Gurrieri C, Capodieci P, Bernardi R, Scaglioni PP, Nafa K, et al. (2004) Loss of the tumor suppressor PML in human cancers of multiple histologic origins. J Natl Cancer Inst 96: 269-279.
22. Plevova P, Bouchal J, Fiuraskova M, Foretova L, Navratilova M, et al. (2007) PML protein expression in hereditary and sporadic breast cancer. Neoplasma 54: 263-268.
23. van 't Veer LJ, Dai H, van de Vijver MJ, He YD, Hart AA, et al. (2002) Gene expression profiling predicts clinical outcome of breast cancer. Nature 415: 530-536.
24. Wieczorek M, Paczkowska A, Guzenda P, Majorek M, Bednarek AK, et al. (2008) Silencing of Wnt-1 by siRNA induces apoptosis of MCF-7 human breast cancer cells. Cancer Biol Ther 7: 268-274.

**Table S2.** The genes and their related Probe Id, q_val is the q_value of gene expression, F_C is Fold-change of gene expression, T_T is T-test value, pt is the p-value of T-test. SAM is the value of SAM (0.3), F_T is the value of F_test, pf is the p-value of F-test for gene expression. *SWang* is the value of *SWang* for gene expression, and P_sw is the p-value of *SWang*.

| **Probe** | **Gene** | **F_C** | **T_T** | **P_T** | **SAM** | **Psam** | **F_T** | **P_F** | ***Swang*** | **P_VAL** |
| --- | --- | --- | --- | --- | --- | --- | --- | --- | --- | --- |
| 204493_at | BID | -0.16 | 0.63 | 0.266 | 0.2602 | 0.398 | 0.386 | 0.54 | 3.7291 | 0.018096 |
| 210185_at | CACNB1 | 0.623 | 1.649 | 0.053 | -0.8164 | 0.7907 | 2.693 | 0.11 | 2.9089 | 0.045287 |
| 205034_at | CCNE2 | 0.286 | 0.844 | 0.202 | -0.3978 | 0.6537 | 0.695 | 0.41 | 3.227 | 0.031636 |
| 211814_s_at | CCNE2 | 0.372 | 1.24 | 0.111 | -0.5561 | 0.7095 | 1.511 | 0.23 | 4.9332 | 0.004946 |
| 213539_at | CD3D | -0.26 | 0.6 | 0.276 | 0.3125 | 0.378 | 0.355 | 0.55 | 2.8725 | 0.047194 |
| 205758_at | CD8A | -0.002 | 0.005 | 0.498 | 0.0025 | 0.499 | 2E-05 | 1 | 3.4476 | 0.024722 |
| 221331_x_at | CTLA4 | -0.48 | 0.996 | 0.162 | 0.5393 | 0.2962 | 0.981 | 0.33 | 4.3671 | 0.009032 |
| 222022_at | DTX3 | 0.028 | 0.056 | 0.478 | -0.0306 | 0.5121 | 0.003 | 0.96 | 3.8221 | 0.016336 |
| 201907_x_at | DVL3 | 0.091 | 0.233 | 0.408 | -0.1166 | 0.5461 | 0.054 | 0.82 | 3.8441 | 0.015946 |
| 205782_at | FGF7 | 0.301 | 0.825 | 0.207 | -0.4027 | 0.6554 | 0.674 | 0.42 | 3.6846 | 0.019008 |
| 215404_x_at | FGFR1 | 0.355 | 1.523 | 0.067 | -0.6065 | 0.7264 | 2.3 | 0.14 | 4.2438 | 0.010317 |
| 208229_at | FGFR2 | 0.337 | 0.826 | 0.207 | -0.4216 | 0.6623 | 0.678 | 0.41 | 3.9495 | 0.014207 |
| 218665_at | FZD4 | -0.04 | 0.166 | 0.434 | 0.0689 | 0.4727 | 0.028 | 0.87 | 4.3872 | 0.008839 |
| 208258_s_at | GAS2L1 | -0.11 | 0.243 | 0.404 | 0.1283 | 0.4493 | 0.06 | 0.807 | 2.8761 | 0.047 |
| 210666_at | IDS | 0.489 | 1.33 | 0.095 | -0.6518 | 0.7411 | 1.755 | 0.192 | 3.3981 | 0.026 |
| 208448_x_at | IFNA16 | 0.694 | 1.551 | 0.064 | -0.8147 | 0.7902 | 2.356 | 0.13 | 3.0662 | 0.037908 |
| 204191_at | IFNAR1 | -0.15 | 0.663 | 0.255 | 0.2599 | 0.3981 | 0.429 | 0.52 | 3.8418 | 0.015986 |
| 205266_at | LIF | -0.12 | 0.271 | 0.394 | 0.1428 | 0.4435 | 0.073 | 0.79 | 3.3339 | 0.028066 |
| 213487_at | MAP2K2 | 0.368 | 1.007 | 0.16 | -0.4968 | 0.6891 | 1.037 | 0.31 | 3.8063 | 0.016622 |
| 209124_at | MYD88 | -0 | 0.037 | 0.485 | 0.0103 | 0.4959 | 0.001 | 0.97 | 3.3569 | 0.027354 |
| 210756_s_at | NOTCH2 | -0.44 | 1.135 | 0.131 | 0.5702 | 0.2857 | 1.294 | 0.26 | 3.3658 | 0.027084 |
| 213945_s_at | NUP210 | 0.084 | 0.361 | 0.36 | -0.8033 | 0.787 | 0.13 | 0.72 | 3.4821 | 0.024 |
| 213040_s_at | NPTXR | 0.689 | 1.501 | 0.07 | -0.1442 | 0.557 | 2.272 | 0.139 | 2.8728 | 0.047 |
| 206880_at | P2RX6 | -0.32 | 0.643 | 0.262 | 0.3519 | 0.3633 | 0.406 | 0.53 | 2.8807 | 0.04676 |
| 204200_s_at | PDGFB | 0.408 | 1.502 | 0.07 | -0.6421 | 0.738 | 2.201 | 0.14 | 3.5593 | 0.021834 |
| 215179_x_at | PGF | 0.46 | 1.606 | 0.058 | -0.7077 | 0.7586 | 2.568 | 0.12 | 3.4319 | 0.025158 |
| 211588_s_at | PML | -0.18 | 0.37 | 0.357 | 0.2024 | 0.4203 | 0.138 | 0.712 | 2.9938 | 0.041 |
| 207499_x_at | UNC45A | 0.177 | 0.441 | 0.331 | 0.4218 | 0.3376 | 0.189 | 0.666 | 2.8475 | 0.049 |
| 210621_s_at | RASA1 | -0.22 | 1.191 | 0.12 | -0.4132 | 0.6593 | 1.451 | 0.23 | 3.1037 | 0.036339 |
| 220738_s_at | RPS6KA6 | 0.328 | 0.819 | 0.209 | 0.7254 | 0.236 | 0.657 | 0.42 | 3.0851 | 0.037108 |
| 210413_x_at | SERPINB4 | 0.054 | 0.156 | 0.438 | -0.0742 | 0.5294 | 0.024 | 0.878 | 3.1612 | 0.034 |
| 212466_at | SPRED2 | 0.235 | 0.555 | 0.291 | -0.2857 | 0.6118 | 0.301 | 0.59 | 4.6338 | 0.00679 |
| 210790_s_at | SAR1A | -0.57 | 1.465 | 0.075 | 0.0497 | 0.4803 | 2.069 | 0.157 | 3.3242 | 0.028 |
| 221554_at | STRADA | -0.03 | 0.119 | 0.453 | -0.2222 | 0.5874 | 0.014 | 0.91 | 2.8479 | 0.048531 |
| 208570_at | WNT1 | -0.27 | 0.543 | 0.295 | 0.2956 | 0.3844 | 0.286 | 0.6 | 4.4085 | 0.008639 |

Table S3. Empirical distribution test for different genes. GENE is the name of relative gene, DATASETs indicate that the data of relative gene are from. KSA is Kolmogorov-Smirnov-Two-Sample Test(Asymptotic), and KA is Kuiper test. In table, Bold and italic rows indicate that the distribution of genes between case and control are different. Row with underline shows the 12 genes in dataset2.

| GENE | DATASETs | KSA[1] | p-value of KSA | KA | p-value of KA |
| --- | --- | --- | --- | --- | --- |
| BCR | Dataset1 | 1.104105 | 0.1745 | 1.656157 | 0.0827 |
| CASP3 | Dataset1 | 0.897085 | 0.3968 | 1.449138 | 0.2219 |
| CCND1 | Dataset1 | 1.173111 | 0.1275 | 1.449138 | 0.2219 |
| EGFR | Dataset1 | 1.207615 | 0.1082 | 1.690661 | 0.0687 |
| IL1R1 | Dataset1 | 1.173111 | 0.1275 | 1.414634 | 0.2560 |
| MCM4 | Dataset1 | 0.931589 | 0.3506 | 1.207615 | 0.5235 |
| MYD88 | Dataset1 | 0.828079 | 0.4992 | 1.656157 | 0.0827 |
| ***PDGFB*** | ***Dataset1*** | ***1.932184*** | ***0.0011*** | ***1.932184*** | ***0.0159*** |
| RRAS2 | Dataset1 | 1.207615 | 0.1082 | 1.414634 | 0.2560 |
| TAGLN | Dataset1 | 0.897085 | 0.3968 | 1.173111 | 0.5753 |
| TP53 | Dataset1 | 1.2076 | 0.1082 | 1.4146 | 0.6250 |
| BCR | Dataset2 | 0.962306 | 0.3126 | 1.378271 | 0.2955 |
| TP53 | Dataset2 | 0.9871 | 0.2840 | 0.9871 | 0.8375 |
| BID | Dataset2 | 1.136142 | 0.1512 | 1.831486 | 0.0303 |
| ***CACNB1*** | ***Dataset2*** | ***1.545898*** | ***0.0168*** | ***1.831486*** | ***0.0303*** |
| ***CCNE2*** | ***Dataset2*** | ***1.558315*** | ***0.0156*** | ***2.123282*** | ***0.0041*** |
| CD3D | Dataset2 | 1.098891 | 0.1786 | 1.651441 | 0.0848 |
| CD8A | Dataset2 | 0.956098 | 0.3201 | 1.415521 | 0.2551 |
| ***CTLA4*** | ***Dataset2*** | ***1.409313*** | ***0.0377*** | ***1.974280*** | ***0.0120*** |
| DTX3 | Dataset2 | 0.974723 | 0.2981 | 1.688692 | 0.0694 |
| DVL3 | Dataset2 | 0.980931 | 0.2910 | 1.688692 | 0.0694 |
| FGF7 | Dataset2 | 1.117517 | 0.1645 | 1.521064 | 0.1615 |
| ***FGFR1*** | ***Dataset2*** | ***1.552107*** | ***0.0162*** | ***1.850111*** | ***0.0270*** |
| FGFR2 | Dataset2 | 1.396896 | 0.0404 | 1.676275 | 0.0742 |
| FZD4 | Dataset2 | 0.918847 | 0.3672 | 1.676275 | 0.0742 |
| GAS2L1 | Dataset2 | 0.894013 | 0.253585 | 1.688692 | 0.0694 |
| ***IDS*** | ***Dataset2*** | ***1.539690*** | ***0.0175*** | ***1.812860*** | ***0.0340*** |
| IFNA16 | Dataset2 | 1.254102 | 0.0861 | 1.682484 | 0.0718 |
| IFNAR1 | Dataset2 | 1.142350 | 0.1470 | 1.843903 | 0.0281 |
| LIF | Dataset2 | 0.956098 | 0.3201 | 1.670067 | 0.0768 |
| MAP2K2 | Dataset2 | 1.353437 | 0.0513 | 1.800444 | 0.0366 |
| MYD88 | Dataset2 | 0.925056 | 0.3591 | 1.819069 | 0.0327 |
| NOTCH2 | Dataset2 | 1.204435 | 0.1099 | 1.508648 | 0.1709 |
| NUP210 | Dataset2 | 1.105100 | 0.1738 | 1.552107 | 0.1396 |
| NPTXR | Dataset2 | 1.403104 | 0.0390 | 1.701109 | 0.0648 |
| P2RX6 | Dataset2 | 1.272727 | 0.0783 | 1.974280 | 0.0120 |
| ***PDGFB*** | ***Dataset2*** | ***1.819069*** | ***0.0027*** | ***2.104657*** | ***0.0048*** |
| ***PGF*** | ***Dataset2*** | ***1.843903*** | ***0.0022*** | ***1.992905*** | ***0.0106*** |
| PML | Dataset2 | 0.918847 | 0.3672 | 1.694900 | 0.0671 |
| UNC45A | Dataset2 | 1.260311 | 0.0834 | 1.825277 | 0.0315 |
| RASA1 | Dataset2 | 0.937472 | 0.3431 | 1.533481 | 0.1524 |
| RPS6KA6 | Dataset2 | 1.136142 | 0.1512 | 1.701109 | 0.0648 |
| SERPINB4 | Dataset2 | 0.713969 | 0.6879 | 1.421730 | 0.2487 |
| SPRED2 | Dataset2 | 1.129934 | 0.1555 | 1.980488 | 0.0115 |
| SAR1A | Dataset2 | 1.285144 | 0.0735 | 2.253659 | 0.0015 |
| STRADA | Dataset2 | 0.856763 | 0.4551 | 1.570732 | 0.1276 |
| WNT1 | Dataset2 | 1.278936 | 0.0759 | 2.129490 | 0.0039 |

Note: The distributions of common genes between Dataset1 and Dataset2 are different, such as TP53, MYM88, PDGFB.

**Reference**

1. Massey FJ (1951) The kolmogorov-smirnov test for goodness of fit. Journal of the American Statistical Association 46: 68–78.
