# Supplementary figures and images for: Systematical Detection of Significant Genes in Microarray Data by Incorporating Gene Interaction Relationship in Biological Systems
Source: PLoS One. 2010 Oct 29;5(10):e13721. doi: 10.1371/journal.pone.0013721 (PMC2966410; doi:10.1371/journal.pone.0013721)

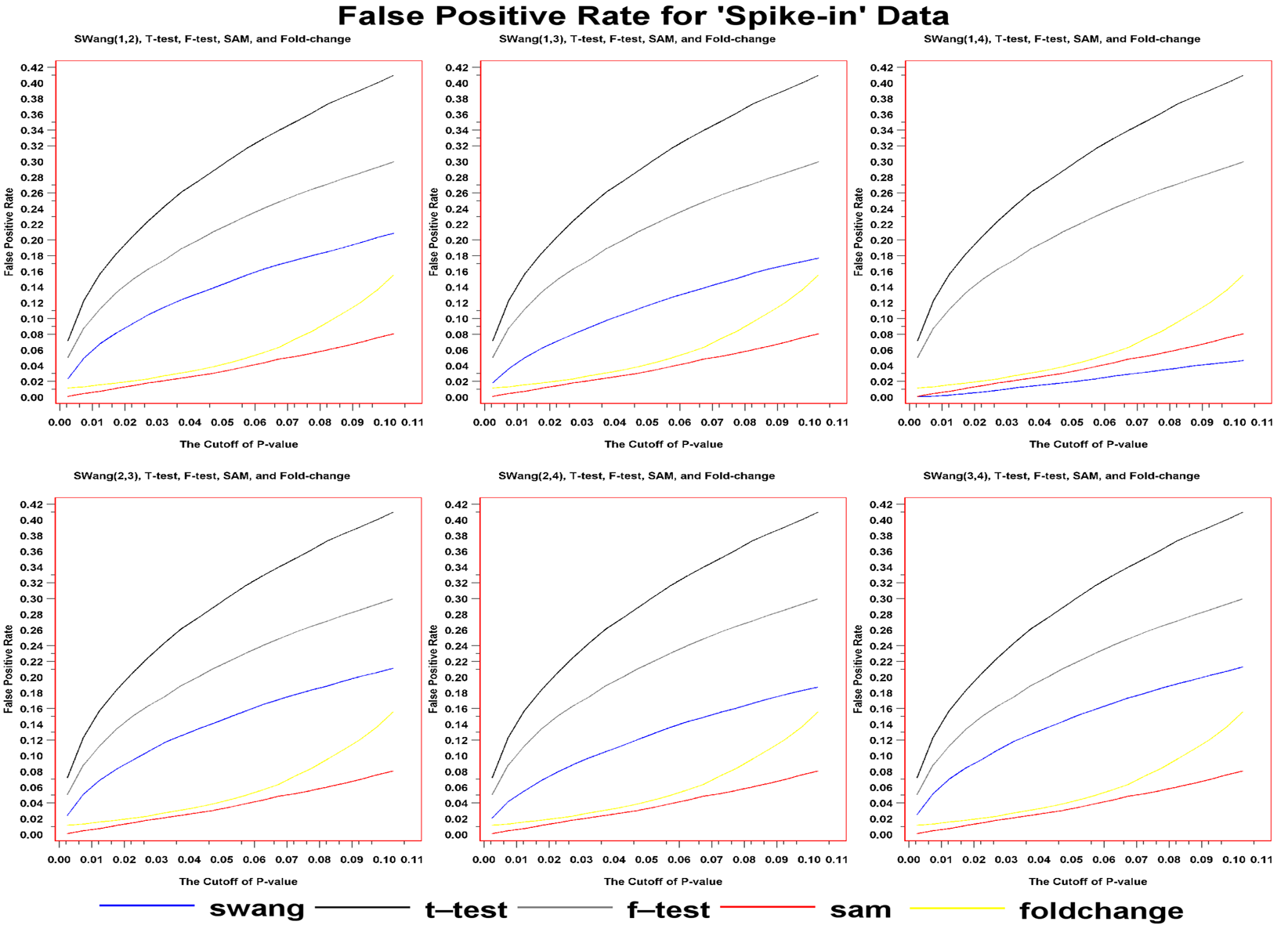

Supplement: Figure S8 — False positive rate of T-test, F-test, Fold-change, SAM(0.3) in ‘Spike-in’ dataset. A: False positive rate of SWang(1,2) and other methods. B: False positive rate of SWang(1,3) and other methods. C: False positive rate of SWang(1,4) and other methods. D: False positive rate of SWang(2,3) and other methods. E: False positive rate of SWang(2,4) and other methods. F: False positive rate of SWang(1,3) and other methods. The false positive rate of T-test(black spotline), F-test(grey spotline), Fold change(yellow spotline), SWang(blue spotline), and SAM(0.3)(red spotline) with cutoff of p-value. (6.41 MB TIF) [file pone.0013721.s013.tif]

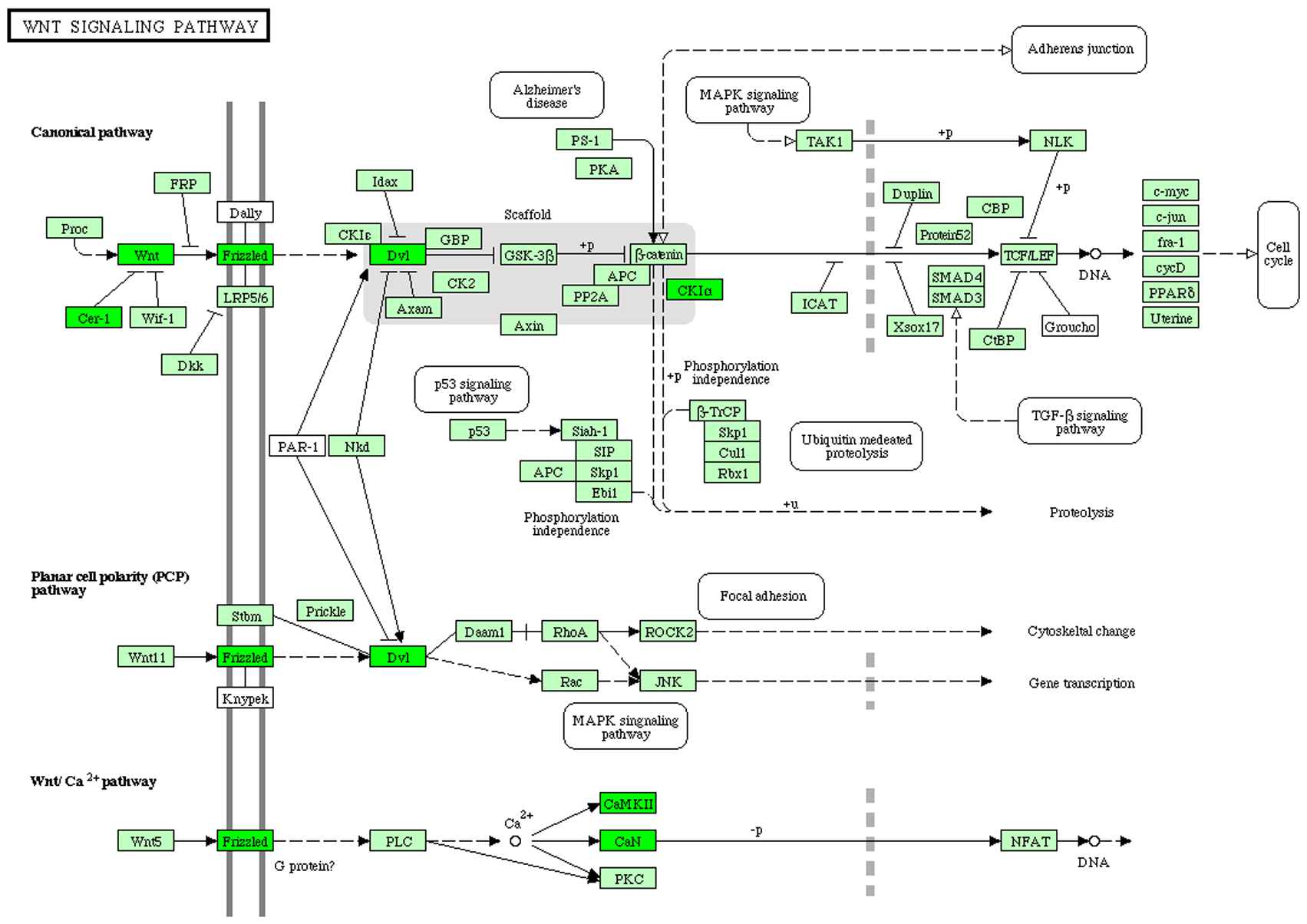

Supplement: Figure S17 — WNT Signal Pathway. The genes detected by SWang test but not by T-test, F-test, Fold-change, and SAM in Wnt signaling pathway based on KEGG. The genes in pink are the genes selected with our method. (6.98 MB TIF) [file pone.0013721.s022.tif]

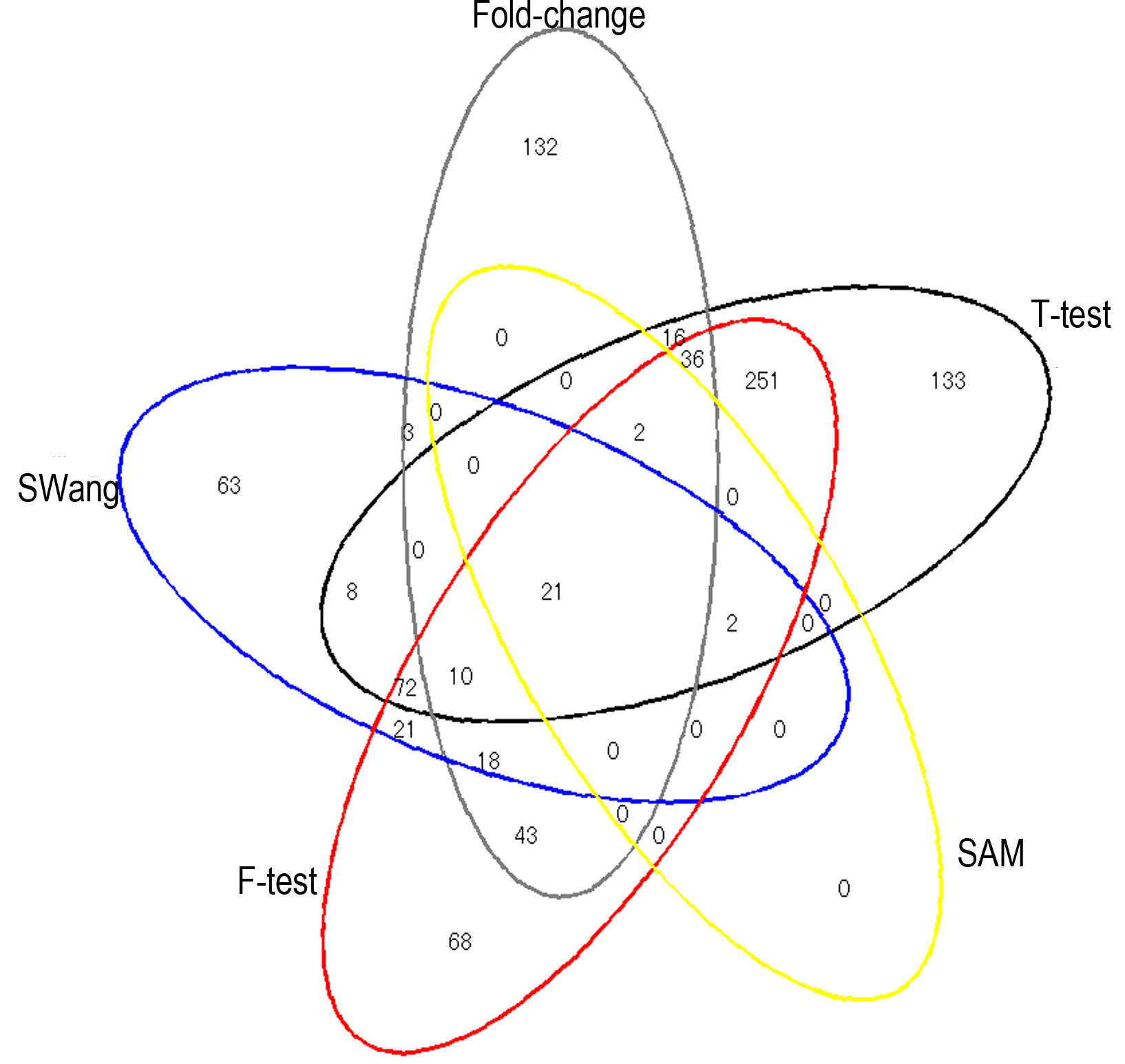

Supplement: Figure S18 — 5-venn diagram in dataset1. The cut-off of p-value of T-test, F-test, SAM(0.3), and SWang is 0.05, the cut-off of Fold-change is 2. (8.71 MB TIF) [file pone.0013721.s023.tif]

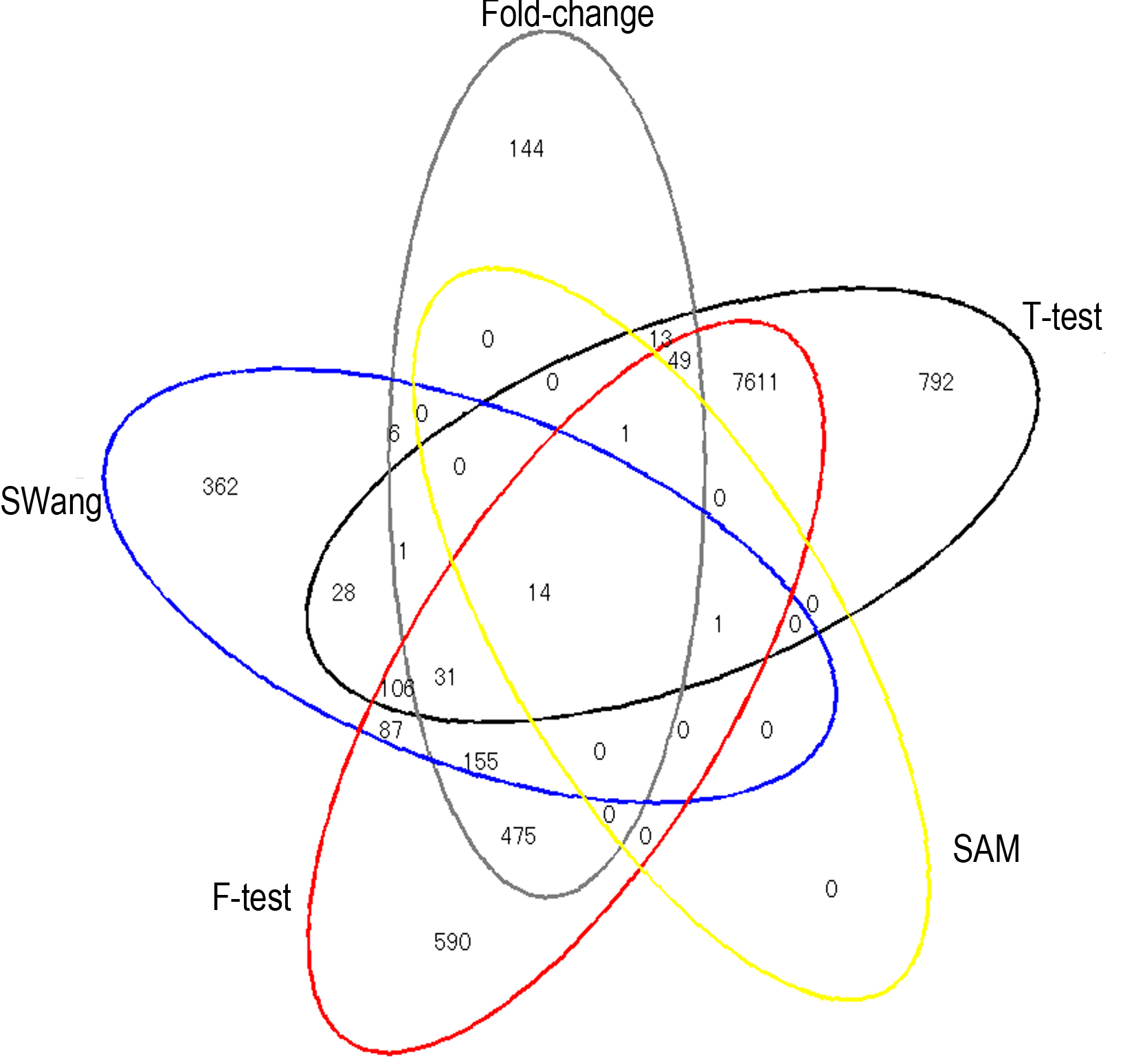

Supplement: Figure S19 — 5-venn diagram in dataset2. The cut-off of p-value of T-test, F-test, SAM (0.3), and SWang is 0.05, while the cut-off of Fold-change is 2. (8.75 MB TIF) [file pone.0013721.s024.tif]

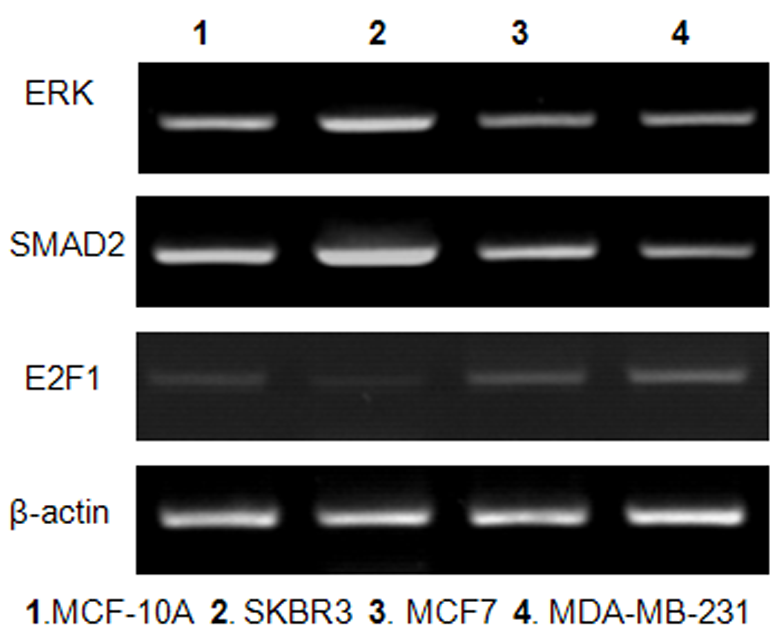

Supplement: Figure S20 — Semiquantative RT-PCR comparision. The genes which could not be detected by Swang test but were by the others are randomly selected. MCF-10A cells were cultured in DMEM/F12 with 10%FBS, 20ng/ml EGF, 0.5ug/ml Hydrocortisone, 0.01ug/ml Insulin and 0.1ug/ml Cholera toxin. MCF-7, SK-BR-3, MDA-MB-453 and MDA-MB-231 were maintained in DMEM with 10%FBS. PCR products were run on 2% agarose gel and then stained with ethidium bromide. Stained bands were visualized under UV light and photographed. The beta-actin used as an internal control. (1.90 MB TIF) [file pone.0013721.s025.tif]
